# Evaluating three alternative scenarios for the origin of a disjunct *Arctium tomentosum* population in the Pyrenees

**DOI:** 10.7717/peerj.21692

**Published:** 2026-09-07

**Authors:** Iván Pérez-Lorenzo, Pol Fernández-Mató, Luis Palazzesi, Anabela Cardoso, Leonardo Platania, Jaume Pellicer, Pere Barnola Echenique, Miquel Àngel Marcé Sallés, Teresa Garnatje, Oriane Hidalgo

**Affiliations:** 1 Botanical Institute of Barcelona, IBB, CSIC-CMCNB, Barcelona, Catalonia, Spain; 2 Botany Laboratory, Faculty of Pharmacy and Food Sciences, University of Barcelona, Barcelona, Catalonia, Spain; 3 Paleobotany Division, Bernardino Rivadavia Argentine Museum of Natural Sciences (CONICET), Buenos Aires, Argentina; 4 Royal Botanic Gardens, Kew, Richmond, United Kingdom; 5 Marimurtra Botanical Garden, Carl Faust Foundation, Blanes, Catalonia, Spain

**Keywords:** *Arctium*, Disjunction, Reproductive biology, Pyrenees, Capitulum traits, Morphology, Genomics

## Abstract

**Background:**

Disjunctions in species distributions offer natural experiments for investigating the mechanisms underlying distributional breaks, such as range contraction, long-distance pollen transport, or long-distance seed dispersal. Here, we document and characterise a floristic novelty in the Pyrenean and Iberian regions, and use it as a case study to investigate the processes underlying disjunct species distributions. Our analysis focuses on *Arctium* L. (burdocks), a genus native to Eurasia comprising several cosmopolitan weedy species. It is characterised by hooked capitula that facilitate epizoochorous dispersal.

**Methods:**

To clarify the taxonomic identity of a previously undocumented Pyrenean population resembling *A. tomentosum*, we adopted an integrative approach, combining distribution information, morphology, genome size, repetitive DNA analysis and chloroplast data across the four known European species. Additionally, we conducted a comparative study of functional capitulum traits, predispersal fruit predation, and associated entomofauna for the newly discovered population and nearby *A. minus* populations.

**Results:**

The discovered population constitutes a floristic novelty for the region and is identified as *A. tomentosum*, exhibiting signatures of past introgression from *A. minus*, as indicated by cytonuclear discordance. We evaluated three alternative scenarios to explain its origin: (i) persistence as a relict from a previously wider distribution, (ii) long-distance pollination by migrating insects, and (iii) long-distance seed dispersal from an *A. tomentosum* population with a history of past introgression. Of these, only the third is consistent with the available evidence. These results highlight the complexity of interpreting disjunct species distributions and underscore the value of integrative approaches for resolving population identity, origin, dispersal, and persistence across environments.

## Introduction

Species distribution disjunctions provide a framework for studying the processes underlying distributional breaks, including long-distance dispersal and range contraction through local extinction (Sanmartín, 2012). Mountain systems are particularly informative in this context because they can influence lineage diversification through multiple non-exclusive mechanisms. In plants, this complexity is reinforced by the fact that movement among populations may occur through pollen and diaspores, and that each pathway can be mediated by a diversity of biotic and abiotic vectors acting over contrasting spatial scales (Robledo-Arnuncio et al., 2014). Mountain ranges may therefore simultaneously restrict connectivity by limiting the movement of propagules (Caplat et al., 2016), while promoting persistence by providing stable microhabitats through climatic oscillations (Selwood & Zimmer, 2020). In this sense, the Pyrenees have acted as long-term refugia retaining relict lineages that were once more widely distributed across Europe (*e.g.*, Gómez & Lunt, 2007). They also function as a semipermeable biogeographic filter, structuring dispersal and gene flow without fully preventing episodes of secondary contact and spatially restricted admixture among previously isolated lineages when climatic and geographic conditions become favourable (Ortego & Noguerales, 2025; Pèlachs et al., 2026). Because these processes are biologically important but often difficult to disentangle, they require study systems in which contrasting historical and ecological scenarios can be evaluated against explicit mechanistic hypotheses.

In this regard, *Arctium* L. (burdocks) may represent a suitable system, and we propose to explore its potential as a case study to investigate the processes underlying disjunct distributions. The genus belongs to Cardueae (Asteraceae) and is placed in the subtribe Arctiinae, a lineage inferred to have originated in the Middle to Late Miocene and comprising *Arctium* and *Cousinia* Cass. (Herrando-Moraira et al., 2019). Burdocks are naturally distributed across temperate Eurasia, with the center of diversity in the mountains of Middle Asia, and are typically associated with open, disturbed, often nutrient-rich habitats. Several species are widespread and behave as ruderal and weedy plants, contributing to their broad distribution beyond their native range (*e.g.*, *Arctium lappa* L. and *Arctium minus* (Hill) Bernh.; Christenhusz, Fay & Chase, 2017). The genus includes taxa of economic and applied interest: *e.g.*, *A. lappa* is cultivated and used as a food, medicinal, and nutraceutical plant (Moro & Clerici, 2021; Song et al., 2023; Yosri et al., 2023), while *Arctium tomentosum* Mill. has been investigated for its potential in metal phytoremediation (Al Harbawee et al., 2017). *Arctium* is also a well-known biomimetic model, given that its hooked involucral bracts inspired the invention of hook-and-loop fasteners (*i.e.,* Velcro; Hunter, 2014). Beyond these aspects, the genus offers a useful system for studying dispersal, adaptation, and species boundaries, particularly in Europe where several closely related taxa coexist (López-Vinyallonga et al., 2011).

Four *Arctium* species are naturally distributed across Europe: *A. lappa*, *A. minus*, *Arctium nemorosum* Lej., and *A. tomentosum*, of which only *A. lappa* and *A. minus* extend into the Pyrenees and the Iberian Peninsula (Ortega, 2014). Although hybrids and introgressants between European species have been reported, gene flow among them appears to be limited (Repplinger et al., 2007). Species delimitation in these closely related taxa is challenging due to morphological overlap and variability in reproductive structures (Duistermaat, 1996), as reflected in their complex taxonomic history. Indeed, *A. minus*, *A. nemorosum* and *A. tomentosum* have at times been treated as infraspecific taxa of *A. lappa*; *A. nemorosum* and *A. tomentosum* have also been considered subspecies of *A. minus* (Plants of the World Online (POWO), 2026). Affinities inferred from morphology suggest a closer relationship between *A. lappa* and *A. tomentosum*, based on their corymbose synflorescences, and between *A. minus* and *A. nemorosum*, based on a combination of traits including synflorescence architecture, capitulum peduncle characteristics, corolla indumentum, and the shape and dimensions of the involucral bracts (Duistermaat, 1996). Relationships remain largely unresolved in molecular studies (Susanna et al., 2003; López-Vinyallonga et al., 2009; López-Vinyallonga et al., 2011; Lavergne et al., 2025). These species are inferred to derive from a lineage of Middle Asia origin (Barres et al., 2013), but the timing of their diversification is still poorly known. One available estimate concerns the divergence between *A. minus* and *A. lappa*, with a mean age of 4.36 Mya in the Pliocene (range: 2.06–7.38 Mya; Barres et al., 2013).

**Figure 1:**
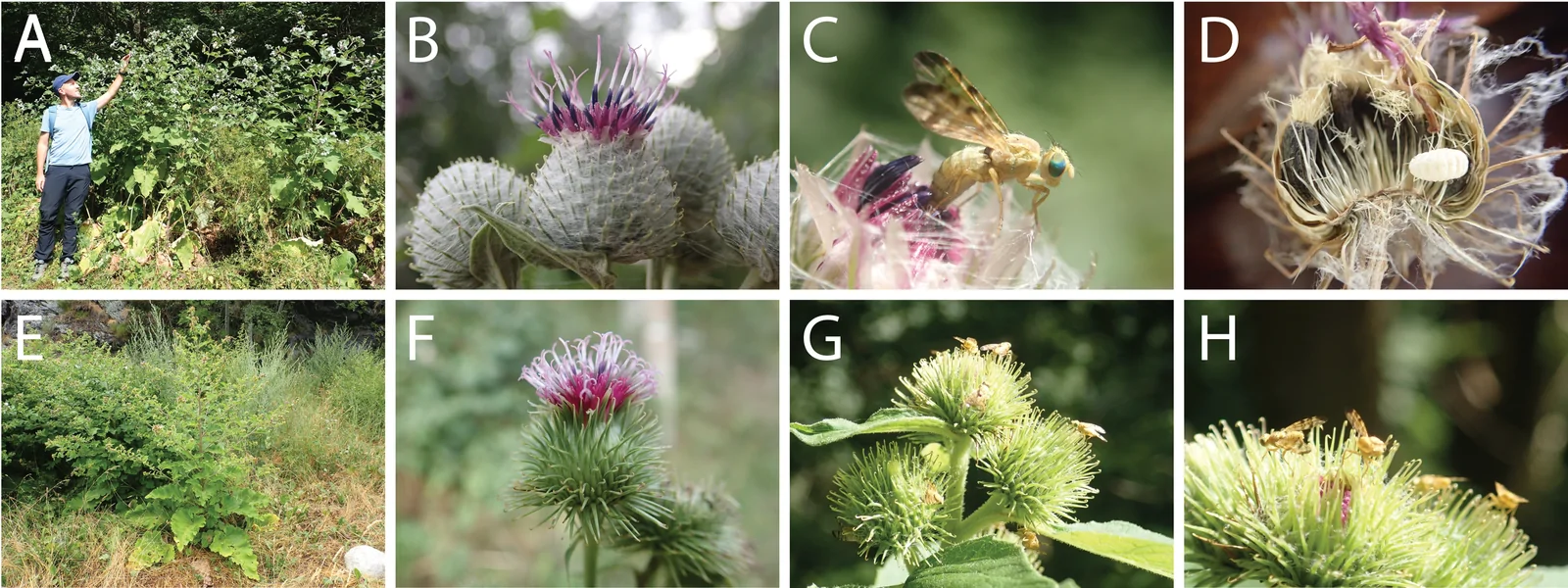
Comparative overview of habit, capitula, and associated fauna in *Arctium tomentosum* and *A. minus*. Images of *A. tomentosum* (A–D) and *A. minus* (E–H), illustrating the general habit of the plants (A, E), the capitulum in lateral view (B, F), and associated fauna with pre-dispersal capitulum predation: tephritid flies ovipositing on a capitulum at anthesis (C) and visiting capitula prior to anthesis (G, H), and a larva of *Metzneria lappella* (Linnaeus, 1758) within a mature capitulum (D).

The discovery in 2021 of a Pyrenean population morphologically consistent with *A. tomentosum* (Fig. 1) is noteworthy, as the species’ southwestern limit was previously restricted to the foothills of the French Alps (Duistermaat, 1996). The occurrence of a natural population of *A. tomentosum* in the Pyrenees would represent the southwestern edge of the species Eurasian range. Based on that, the main objective of this study is to investigate the origin of this disjunct population, as well as the processes and mechanisms underlying its presence and persistence, by exploring alternative evolutionary scenarios. To this end, we integrated morphological, genomic, and cytogenetic data in a comparative framework to assess the taxonomic identity of the Pyrenean population and its affinities with closely related species. We also examined, at a local scale, different aspects of its reproductive biology.

## Materials & Methods

### Study design

We adopted an integrative approach combining occurrence synthesis, herbarium-based comparative morphology, genomic, and reproductive success studies. We compiled and filtered occurrence records from the Global Biodiversity Information Facility (GBIF), searching for accessions attributable to *A. tomentosum* in the Pyrenees. In addition, BC and JACA herbaria were examined to look for potential Pyrenean specimens of *A. tomentosum* that might still be undigitized; this herbaria audit also provided the curated material used for the comparative morphological study (Table S1).

For genome size estimation, individuals were sampled from populations in the surroundings of Setcases (Girona, Catalonia, Spain): one individual of *A. tomentosum* (population OH 775) and of *A. lappa* (OH 833), and two individuals of *A. minus* (OH 728 and OH 779; Table S2). For comparative purposes, unpublished genome size data from Alpine accessions were also used: *A. lappa* (4.09 pg/2C), *A. minus* (4.61 pg/2C), *A. nemorosum* (4.83 pg/2C) and *A. tomentosum* (3.70 pg/2C, OH 476; L Pegoraro, 2026, unpublished data).

Genome skimming was performed on the same set of individuals used for genome size estimation, except for OH 779, and included an additional *A. tomentosum* individual collected in the Slovenian Alps (OH 476). Genomic data from these Pyrenean accessions were analysed for repeatome composition and phylogenetic relationships, together with data from Alpine accessions generated by the PhyloAlps project (Lavergne et al., 2025). The Alpine dataset included one individual each of *A. lappa*, *A. minus*, and *A. nemorosum*, and two individuals of *A. tomentosum* (Table S3), providing a broader comparative framework. *Cousinia* accessions from Karimov et al. (2025) and Kenesbay et al. (2025) were used as outgroups in the phylogenetic reconstructions (Table S3).

The reproductive success analysis was based on the same *A. minus* and *A. tomentosum* populations from Setcases used in the cytogenetic survey, and additionally included two nearby populations of *A. minus* (OH 777 in Setcases and OH 951 in Llanars). Sampling was conducted during the summers of 2022 and 2023 (July–September; Table S4), constrained by extreme drought in 2022 and by the combined effects of herbivory and vegetation management in 2023. Only terminal capitula from the main (primary and secondary) axes were collected. Fresh, flowering terminal capitula at comparable phenological stages were sampled to characterize capitulum functional traits (Table S5). Mature terminal capitula were collected to quantify flower-to-fruit conversion and estimate predispersal reproductive success. Insects found within mature capitula were preserved in absolute ethanol for subsequent morphotype classification and molecular barcoding (Table S6). Herbarium vouchers resulting from field collections were deposited in BC herbarium.

### European distribution of *A. minus* and *A. tomentosum*

Occurrence records from *Arctium* were downloaded from GBIF (accessed on 24/03/2026; DOI: https://doi.org/10.15468/dl.umhpwn). To compile the distribution map of *A. minus* and *A. tomentosum*, records were filtered to ensure quality and verifiability (Zizka et al., 2020; López-Guillén et al., 2024): (i) selecting only the observations corresponding to these two taxa; (ii) only herbarium specimens or citizen-science iNaturalist observations were retained basisOfRecord = PRESERVED_SPECIMEN, and HUMAN_OBSERVATION records only when publisher = iNaturalist; (iii) citizen records had to be recent (year ≥ 2000) with coordinate uncertainty ≤ 100 m; and (iv) observations had to fall within the period when *Arctium* is most likely to bear reproductive structures (July–September). Then we retained only occurences from Europe. The subset of *A. tomentosum* records falling within the Iberian Peninsula (Spain and Portugal), the Balearic Islands, and the Pyrenees (southern France and northern Spain) was also manually inspected prior to filtering (Table S7). Maps were produced in R (R Core Team, 2026) using the packages sf (WGS84; EPSG:4326; Pebesma, 2025), ggspatial for plotting (Dunnington, 2025) and rnaturalearth (South, 2017) for country boundaries.

### Morphological studies in herbarium specimens

Twenty-two herbarium specimens from Europe, including the Pyrenean vouchers for the genomic studies, were selected based on the presence of all relevant quantitative and qualitative traits for species diagnosis, optimal preservation, and comparable phenological stages (Table S1). These included *A. minus* (seven specimens), *A. tomentosum* (four specimens), *A. lappa* (five specimens), and their respective hybrids (six specimens). The sampling encompasses all *Arctium* species currently known from the Pyrenees (*A. minus* and *A. lappa*), as well as *A. tomentosum*, the species to which the newly identified Pyrenean population is morphologically most similar. Nine quantitative traits and five qualitative traits were assessed from the three taxonomic sources consulted (Perring, 1976; Duistermaat, 1996; Ortega, 2014). The quantitative traits comprised peduncle length; involucrum diameter and height; involucrum diameter and height excluding the apical portion of the bracts; and the length and width of the middle and inner bracts. The qualitative traits, selected for their diagnostic value, included flower position relative to the involucral bracts, indicating whether the flowers extend beyond or remain below them (binary), inner-bract apex shape (ordinal), involucrum pubescence (ordinal), corolla indumentum (ordinal), and synflorescence type (nominal). Data processing followed a mixed-variable framework: quantitative variables were kept as numeric; the binary trait was encoded as presence/absence (0/1); ordinal traits were coded as ordered factors with increasing levels (0 < 1 < 2); and the nominal trait was coded as an unordered factor. We computed pairwise dissimilarities with Gower’s distance (Gower, 1971) using daisy function (metric = “gower”) from the cluster package in R (Maechler et al., 2025), explicitly declaring the binary and ordinal variables to preserve their scale and ranking. Character states are detailed in Table S1. Quantitative variables were not pre-standardized since Gower internally standardizes them. The matrix was imported to SplitsTree App (Huson & Bryant, 2024) and represented as a neighbor-net (Bryant & Moulton, 2002).

### Genome size estimation

Genome size was estimated from fresh leaf material using propidium iodide flow cytometry with a CyFlow Space (Sysmex-Partec, Germany), following the one-step procedure (Doležel, Greilhuber & Suda, 2007) with modifications as described in Clark et al. (2016). We used *Pisum sativum* L. ‘Ctirad’ (9.09 pg/2C; Doležel, Sgorbati & Lucretti, 1992) as calibration standard and the general purpose buffer (GPB; Loureiro et al., 2007) supplemented with 3% PVP-40.

### Genomic analysis

Genomic DNA extraction was done from silica-dried material using the standard CTAB protocol (Doyle & Doyle, 1987). Libraries of 500 bp fragments were prepared and sequenced at Macrogen on an Illumina NovaSeq 6000 platform generating 150 bp paired-end reads. Read quality was assessed using FastQC (Andrews, 2010), and sequences were preprocessed with fastP (Chen et al., 2018) to remove adapters and trim high quality reads to 120 bp.

The resulting dataset was used to characterize genome composition and repetitive DNA content with RepeatExplorer2 (Novák, Neumann & Macas, 2020). Both individual and comparative analyses were performed. Individual analyses were conducted using the maximum number of reads supported by the available computational resources (representing approximately 6.7% of each genome), whereas the comparative analysis included a reduced number of reads, with each species contributing ∼0.6% of its genome. Automatic annotation of repetitive elements from individual analyses was manually curated. Using the comparative dataset (Table S8), the number of copies of each cluster per individual were compared using linear regressions. Higher pairwise coefficients of determination (R^2^) indicate greater similarity among samples in their repetitive DNA landscape.

The dataset was also used to infer nuclear and plastid phylogenies. The 45S ribosomal RNA region (including ITS1 and ITS2) was extracted for each individual as a consensus sequence from the RepeatExplorer2 analysis. For each species, all reads were mapped to the consensus sequences, applying a 95% similarity threshold for each locus. Absence of gene variants for the 45S loci was confirmed by manually inspecting RepeatExplorer graphs. The resulting sequences were then aligned using MAFFT (Katoh & Standley, 2013) with default parameters, and a phylogenetic tree was constructed using IQ-TREE v 2.4 running model finder and a non-parametric bootstrap (BS) of 1,000 replicates (Minh et al., 2020). Partial plastomes were reconstructed by mapping raw reads to a consensus sequence derived from published complete plastomes using Geneious 2025.2.2 (Biomatters Ltd., Auckland, New Zealand). A consensus sequence was generated with a 95% similarity threshold. In addition, de novo plastome assembly was performed with NOVOPlasty (Dierckxsens, Mardulyn & Smits, 2017), yielding scaffolds that were subsequently mapped to the reference consensus to identify potential structural variants. Sequence alignment and phylogenetic reconstruction for the plastome dataset were conducted following the same procedure described above for the 45S dataset.

### Functional capitulum traits

In 2023, 52 fresh flowering capitula were sampled from intact individuals (without symptoms of cutting or predation) from three populations of *A. minus* (*n* = 27) and the population of *A. tomentosum* (*n* = 25), and processed on the same day (Table S5). Due to limited availability of intact *A. minus* individuals across sites, samples from the three populations were pooled for statistical comparisons to ensure a sample size comparable to *A. tomentosum*. The following morphological traits were recorded: capitulum height, involucrum height and diameter, and number of bracts and flowers.

Descriptive statistics were computed in R (R Core Team, 2026). Given the small sample size and the comparison of only two species, we evaluated between-species differences with the non-parametric Wilcoxon rank-sum test (wilcox_test in rstatix; Kassambara, 2023). Associations among traits were assessed with Spearman’s rank correlations “*ρ*” using cor and cor.test functions on log-transformed variables. After confirming that any variable correlation did not exceed 0.90, we performed a Principal component analysis (PCA) on the log-transformed traits with prcomp function (centred and scaled). For visualisation, we plotted pairwise relationships among traits using a scatterplot matrix, the first two principal components and a correlation unit circle for the eigenvectors with ggplot2 (Wickham et al., 2019).

### Predispersal reproductive success

During 2022 and 2023, 131 capitula with mature fruits but still retaining the dry flowers were sampled from four *A. minus* (*n* = 74) and *A. tomentosum* (*n* = 57) populations (Table S4). Capitula were dissected to count the number of flowers and fruits, discarding any capitulum in which the number of fruits exceeded the number of flowers, as this indicates flower loss prior to collection. Fruits were then classified into three mutually exclusive categories: well-formed, aborted, or predated. In a subset of capitula, the number of flowers exceeded the number of fruits recovered. To account for this, analyses of predated fruits were conducted twice: (i) using the recorded counts and ignoring the discrepancy (“without”), and (ii) treating the missing portion as fully predated cypselae, and therefore adding that difference to the number of predated fruits (“with”).

For each capitulum and fruit category, empirical conversion rates were calculated as the percentage of flowers assigned to that category. These ratios were used to ensure comparability among capitula despite natural variation in the number of flowers. Complementarily, flower-to-fruit conversion was analysed with generalized linear mixed models (GLMMs), with species as a fixed effect and *A. minus* as the reference level. Variance partitioning across population, year, and their interaction was first assessed from models including these random effects (Nakagawa, Johnson & Schielzeth, 2017), and the results were used to define the random-effects structure of the final GLMMs.

We first analysed each fruit category using binomial models at the capitulum level. Overdispersion was assessed with the check_overdispersion function in the performance package (Lüdecke et al., 2021), and was detected in the models for well-formed and aborted fruits. We therefore compared binomial and β-binomial models using Akaike’s Information Criterion (AIC; Symonds & Moussalli, 2011). As β-binomial models consistently showed a better fit, all final models were fitted with a β-binomial distribution. Model adequacy was then evaluated in DHARMa using 1,000 simulated residuals and tests of uniformity, dispersion, and zero inflation (Hartig, 2024).

Mean differences in empirical conversion rates were assessed in R using Wilcoxon rank-sum tests, both in a global comparison between species and in a complementary set of separate contrasts to evaluate temporal and spatial variation among years, months and populations, with *p* values adjusted using the Benjamini–Hochberg false discovery rate procedure. Mean differences in the fitted models were evaluated with the emmeans package in R (Lenth, 2025). Additionally, two complementary regressions were conducted using the lm function in R. First, reproductive investment was examined by fitting a linear model of well-formed fruits per capitulum as a function of flowers per capitulum. Second, the relationship between predation and abortion was assessed with a linear model using a compositional approach (Aitchison, 1982), in which predated and aborted fruits were expressed as log-ratios relative to well-formed fruits. All graphics were produced with ggplot2 (Wickham et al., 2019).

### Insect sampling and identification

All arthropods found during the dissections of mature capitula of *A. minus* and *A. tomentosum* during the 2023 campaign were collected and preserved in absolute ethanol. Sampling included three nearby populations: (i) *A. tomentosum* (21 capitula; OH 775) at the edge of a path that crosses a small grove, sampled in July and September; (ii) a forest population of *A. minus* (12 capitula; OH 777), sampled in August; and (iii) a second population of *A. minus* (26 capitula; OH 951) near orchards, in a more open environment, also sampled in August (Table S6). To guide molecular identification, insects were first assigned to morphogroups based on developmental stage (larva or adult) and taxonomic order, with each distinct morphology defining a separate group. For each plant species, population, and sampled month, one representative individual of each morphogroup was selected for sequencing, in order to capture taxonomic, functional, and spatio-temporal diversity. DNA was extracted using the DNeasy Blood and Tissue kit (Qiagen Iberia, Madrid) and all samples were processed using non-destructive methods following the kit instructions with minor modifications. DNA samples and voucher specimens are deposited in the Replace with Botanical Institute of Barcelona (IBB) entomological collection. A fragment of the mitochondrial gene Cytochrome C oxidase subunit I (5′ end), the standard barcode region (Hebert, Ratnasingham & De Waard, 2003), was amplified using primers LCO1490 and HCO2198 (Folmer et al., 1994). For specimens with difficult amplification, primers UNILEPF1B and UNILEPR1 were used (Hebert et al., 2004). PCR products were sequenced in both directions using BigDye Terminator 3.1 chemistry on a 3730xl DNA Analyzer (Applied Biosystems, Foster City, USA). The resulting sequences were edited in Geneious Prime 2025.2.2 (Biomatters Ltd., Auckland, New Zealand) and identified using the Barcode ID function on the “Animal library” dataset in the Barcode of Life Data System (BOLDv5; Ratnasingham & Hebert, 2007), retaining only matches with ≥ 94% similarity. Descriptive statistics combining molecular identification and morphogroups-based counts were calculated using basic R functions.

## Results

### Distribution of *A. minus* and *A. tomentosum* and the southwestern range limit of *A. tomentosum*

**Figure 2:**
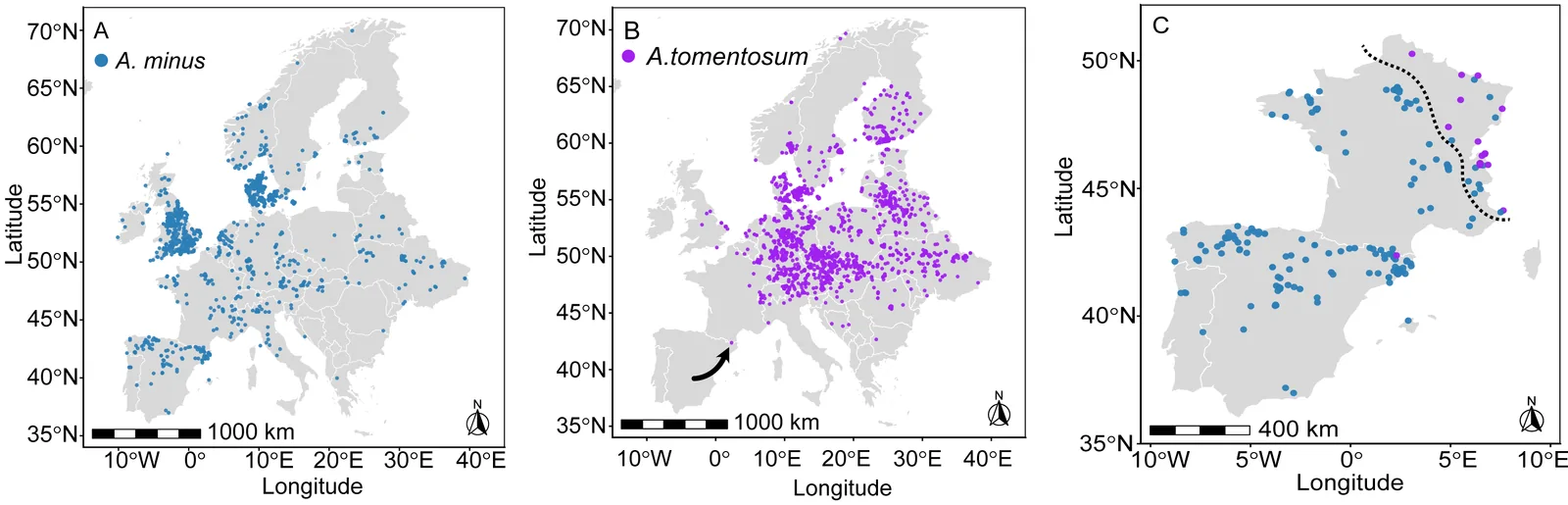
Distribution maps based on filtered GBIF data of *A. minus* (A) and *A. tomentosum* (B) across Europe. The black arrow in (B) indicates the location of the Pyrenean population. (C) shows a detailed view of the distribution of both species in France, the Iberian Peninsula and the Balearic Islands, highlighting the southwesternmost limit of the known range of *A. tomentosum* with a dashed line. Maps were generated in R using basemap vector data from Natural Earth, accessed through the rnaturalearth package. The dashed line was manually added using Affinity Designer. The filtered occurrence dataset containing the points used to generate the distribution maps is available at https://doi.org/10.5281/zenodo.20489435. No external templates or silhouettes were used in this figure.

GBIF searches, after taxon selection, spatial delimitation, and data quality filtering, yielded 1,444 and 2,175 occurrence records for *A. minu* s and *A. tomentosum*, respectively, which were used to illustrate their European distributions (Fig. 2). All 28 occurrence records of *A. tomentosum* retrieved from GBIF within the Iberian Peninsula, Balearic Islands, and Pyrenees failed automated quality filters, indicating potential issues with data quality, georeferencing, and/or traceability (Table S7). A manual review revealed that 11 of these observations were located in the Botanical Garden of the University of Coimbra (Portugal) between the years 1953 and 1976, suggesting that these specimens were cultivated. Twelve records were obtained from the Pl@ntNet platform including eight with associated images, either misidentified or with insufficient information for species-level identification, considered to be erroneous; the remaining four lacked images and were considered unverifiable. Four records from Mallorca (Balearic Islands) comprised one fruit accession (unverifiable), one misinterpreted citation from De Bolòs (1996), which mentions only *Arctium chabertii* Briq. & Cavillier (synonym of *A. minus*), and two herbarium specimens with notation errors. Finally, one record was an untraceable human observation from Bayonne (France). After filtering, the only retained record is the *A. tomentosum* from the Pyrenees reported in the present study. Except for our sampling, none of the specimens preserved in the BC and JACA herbaria collected from the Pyrenees correspond to *A. tomentosum*. The species has no known historical records across the Pyrenees; the only previous reported occurrence, from the northeastern Pyrenees and based on a late 19^th^-century literature citation, is considered probably erroneous (Ortega, 2014). Therefore, to our knowledge, the discovered *A. tomentosum* population represents the first verifiable record of a natural population in the Pyrenees, the Iberian Peninsula, and the Balearic Islands.

### Morphological affinities of the disjunct population based on herbarium vouchers

Quantitative and qualitative morphological assessments of the herbarium material (Table S1) showed that the specimen from the disjunct Pyrenean population clusters with other European *A. tomentosum* specimens in the distance network (Fig. S1).

### Genomic affinities of the disjunct population

**Figure 3:**
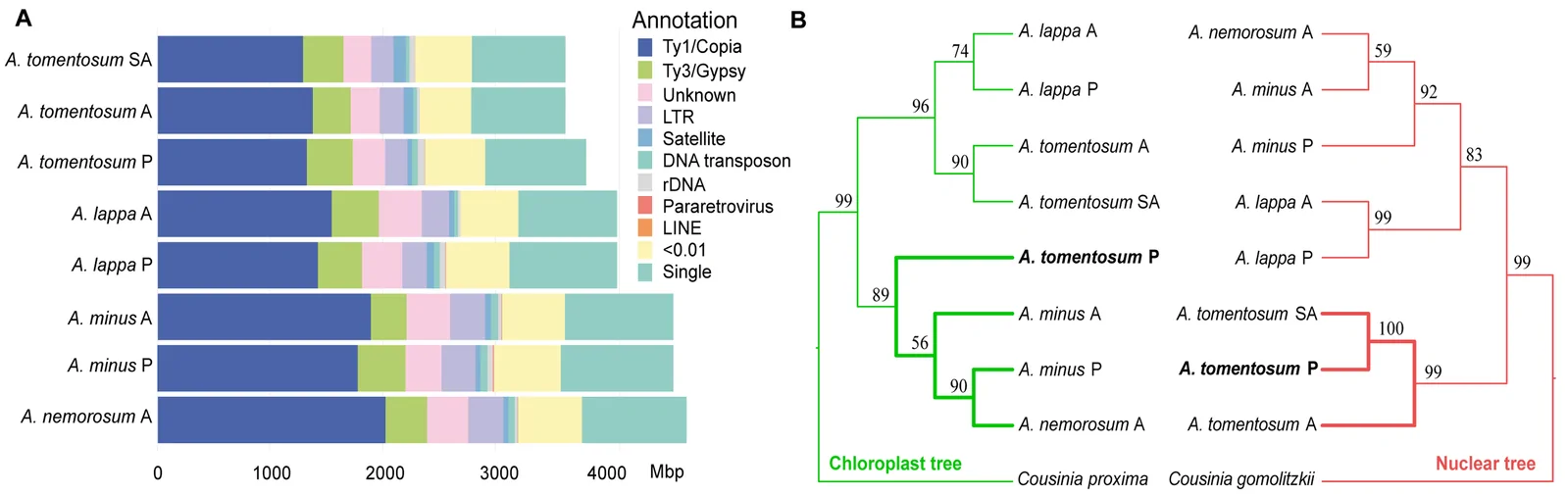
Comparative genomic characterization of *Arctium* accessions. (A) Annotation of repetitive elements across the analysed genomes, shown together with 2C genome size in megabase pairs (978 Mbp ≈1 pg DNA according to Doležel et al., 2003). (B) Chloroplast phylogeny (left) and nuclear phylogeny (right) of *Arctium* accessions, with bootstrap support values indicated at the nodes. Letters following species names indicate geographic origin: P, Pyrenees; SA, Slovenian Alps; A, the rest of the Alps (see also Table S3 for more details).

*Arctium* accessions from the Pyrenees showed different genome size values, *A. minus* 4.67 pg/2C, *A. lappa* 4.17 pg/2C, *A. tomentosum* 3.89 pg/2C (Fig. 3, Table S2). They are consistent with the values reported for corresponding Alpine populations (see ‘Study design’; Fig. 3A).

Analysis of the repeatome showed that the overall composition and relative abundance of repetitive elements are extremely similar across individuals (Fig. 3A; Table S9). R^2^ of repeat abundances were uniformly high (*R*^2^ = 0.925–0.998; Table S9), with the strongest similarities within species. In particular, *A. tomentosum* from the Pyrenees and the Slovenian Alps showed an almost perfect correlation (*R*^2^ = 0.998), and Pyrenean and Alpine populations of *A. lappa* and *A. minus* also displayed very high within-species correlations (*R*^2^ = 0.986 and *R*^2^ = 0.979, respectively). Among species, *A. minus* and *A. nemorosum* were especially similar (*R*^2^ = 0.986 and *R*^2^ = 0.996), while *A. lappa* and *A. tomentosum* also showed high correlations (*R*^2^ = 0.975 and *R*^2^ = 0.985).

The plastid and nuclear phylogenies recovered in Fig. 3B highlighted clear cytonuclear discordance in the placement of the Pyrenean *A. tomentosum* sample. In the nuclear tree, the Pyrenean accession was unambiguously embedded within *A. tomentosum*, forming a strongly supported subclade with the Slovenian-Alpine sample (BS = 100%), and together with the remaining *A. tomentosum* accession a well-supported clade (BS = 99%). In contrast, the plastid tree placed the Pyrenean *A. tomentosum* in a different lineage: rather than clustering with the other *A. tomentosum* samples, which group with *A. lappa* (BS = 96%), it is recovered in a clade with the *A. minus*–*A. nemorosum* assemblage (BS = 89%).

### Morphospace segregation and key capitulum traits

**Figure 4:**
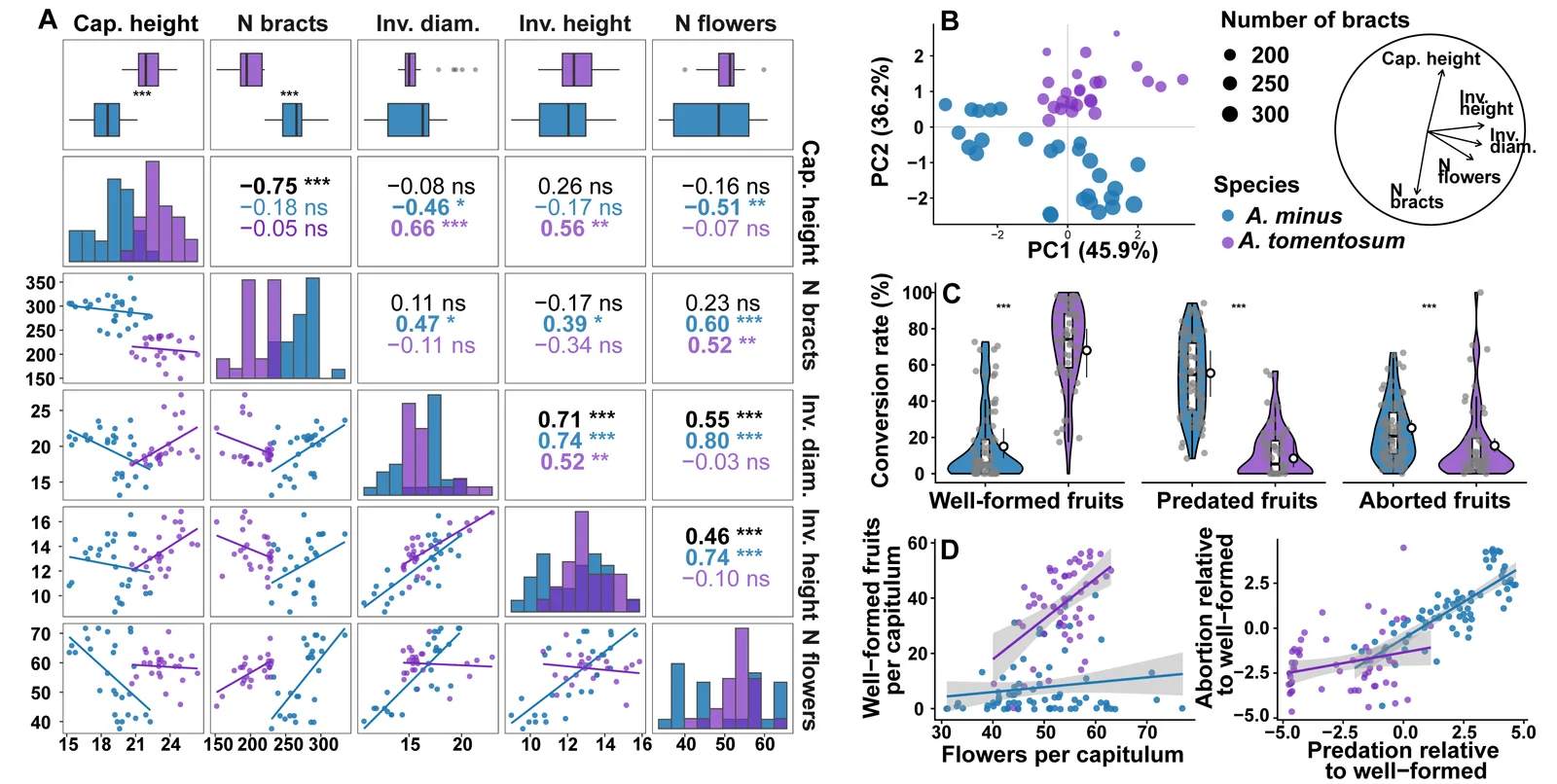
Functional trait differentiation and reproductive outcomes in *A. minus* and *A. tomentosum*. (A) Scatterplot matrix of five capitulum traits measured in 27 capitula of *A. minus* (blue; three populations pooled) and 25 of *A. tomentosum* (purple). Diagonal panels show trait distributions, upper panels interspecific comparisons, and lower panels pairwise relationships with Spearman’s correlations calculated jointly and by species; asterisks indicate significant differences (ns = not significant; *, *p* < 0.05; **, *p* < 0.01; ***, *p* < 0.001; Wilcoxon rank-sum test). (B) PCA of the five traits, with individuals coloured by species and point size proportional to the number of involucral bracts; the inset shows trait loadings. (C) Conversion rates of well-formed, predated, and aborted fruits. Violin plots, boxplots, and points represent empirical capitulum-level data, whereas circles and error bars show GLMM estimates and confidence intervals. Fully predated fruits were calculated as the difference between flower number and recovered fruits under the “with” scenario. Interspecific differences were significant for all fruit categories (*p* < 0.001) in both empirical and model-based analyses and under both predation codings. (D) Relationships between reproductive components at the capitulum level: well-formed fruits *versus* flower number (left), and abortion *versus* predation expressed as log-ratios relative to well-formed fruits under the “with” scenario (right). Lines represent fitted linear models for each species. See ‘Predispersal reproductive success’ and Table S16 for further details.

Analysis of morphological data obtained from fresh capitulum dissections (Fig. 4, Table S5) showed that *A. minus* and *A. tomentosum* occupy distinct, non-overlapping regions of morphospace (Fig. 4B). Most of this species-level differentiation was explained by two functional traits (Fig. 4A, Table S10): *A. tomentosum* had taller capitula than *A. minus* (23.4 ± 1.45 *vs.* 19.0 ± 1.88 mm, *p* < 0.001), whereas *A. minus* bore more involucral bracts (275.4 ± 24.2 *vs.* 205.0 ± 22.7, *p* < 0.001). Reflecting this interspecific difference, capitulum height and bract number were strongly negatively correlated when both species were pooled (*ρ* = −0.75, *p* < 0.001), but not within either species separately (Fig. 4A).

### Patterns of flower-to-fruit conversion rates

Analysis of the flower counts and fruit-classification data (Table S4) showed that variance partitioning in the production of well-formed fruits was almost entirely (99%) explained by year (Table S11). For predated fruits, most of the explained variance was associated with population (90.45% when fully predated fruits were excluded; 70.45% when they were included), with year accounting for the remaining share (9.54% and 29.54%, respectively). For aborted fruits, the random effects did not explain any proportion of variance. Accordingly, the final models included species as a fixed effect in all cases, with year fitted as a random intercept for well-formed fruits, both population and year fitted as random intercepts for predated fruits under both coding schemes, and no random effects included for aborted fruits.

After confirming the suitability of the β-binomial distribution (Table S12), we tested species differences in fruit fate using GLMMs for the three fruit categories under the two predation codings (Table S13). Using *A. minus* as the reference level, species effects on the logit scale were significant in all models, indicating that *A. tomentosum* had a higher probability of producing well-formed fruits and lower probabilities of aborted and predated fruits. This pattern was consistent in both the empirical comparisons and the model-based estimates (Table S14, Fig. 4C), with *A. tomentosum* showing a much higher proportion of well-formed fruits than *A. minus* (69.8% *vs.* 15.2%) and markedly lower proportions of predated fruits under both codings (11.3% *vs.* 53.8%, and 14.5% *vs.* 61.0%, respectively), whereas differences in aborted fruits were smaller but still significant (15.7% *vs.* 23.9%). Model-predicted means closely matched the empirical values, indicating that the GLMMs captured the main biological pattern while accounting for population- and year-level variation. Consistently, analyses across comparison schemes showed that species differentiation was stronger than temporal or spatial variation, as most consecutive-year and among-population contrasts were non-significant (Fig. S3, Table S15).

The results of the linear regressions (Fig. 4D; Table S16) showed that floral production translated into a higher number of well-formed fruits in *A. tomentosum*, but not in *A. minus*. Additive log-ratio models further indicated a strong and consistent positive association between predation and abortion in *A. minus*, regardless of whether fully predated fruits were excluded or included, whereas in *A. tomentosum* this relationship was much weaker and depended on predation coding.

### Entomofauna in *Arctium* heads

During the dissection of 59 mature capitula of *A. minus* (*n* = 38) and *A. tomentosum* (*n* = 21), 265 insects were recovered (226 larvae and 39 adults) and classified into nine morphogroups (Table S6). DNA barcoding (File S1) indicated that these morphogroups correspond to eight taxa (Table S6): the tephritid flies *Terellia tussilaginis* (Fabricius, 1775) (Diptera larvae 1 & 2) and *Tephritis bardanae* (Schrank, 1803) (Diptera adult 1), *Orius laevigatus* (Fieber, 1860) (Hemiptera adult 1) and *Anthocoris nemorum* (Linnaeus, 1761) (Hemiptera adult 2), *Metzneria lappella* (Lepidoptera larvae 1), a Pteromalid wasp (Hymenoptera parasitoid adult 1) *Bracon* Fabricius, 1804, sp. (Hymenoptera parasitoid adult 2), and *Corticaria obscura* Brisout*,* 1863 (Coleoptera adult 1). Barcoding results were consistent across morphogroups, except for two cases that required minor adjustments. Molecular identification of Diptera larvae 1 and 2 corresponded to *Terellia tussilaginis*; however, only a small subset was sequenced, and an adult *Tephritis bardanae* was also recovered. As larvae of these tephritids cannot be reliably distinguished morphologically, we pooled Diptera larvae 1 and 2 for subsequent analyses and attributed both species to this morphogroup. Amplicons obtained from the morphogroup “Hymenoptera parasitoid adult 1” matched an endosymbiotic bacterium of the genus *Wolbachia* Hertig, 1936, associated with different pteromalid wasps. Because the external morphology of our specimens was consistent across individuals and coherent with a pteromalid wasp, we conservatively accepted the identification as Pteromalidae.

**Figure 5:**
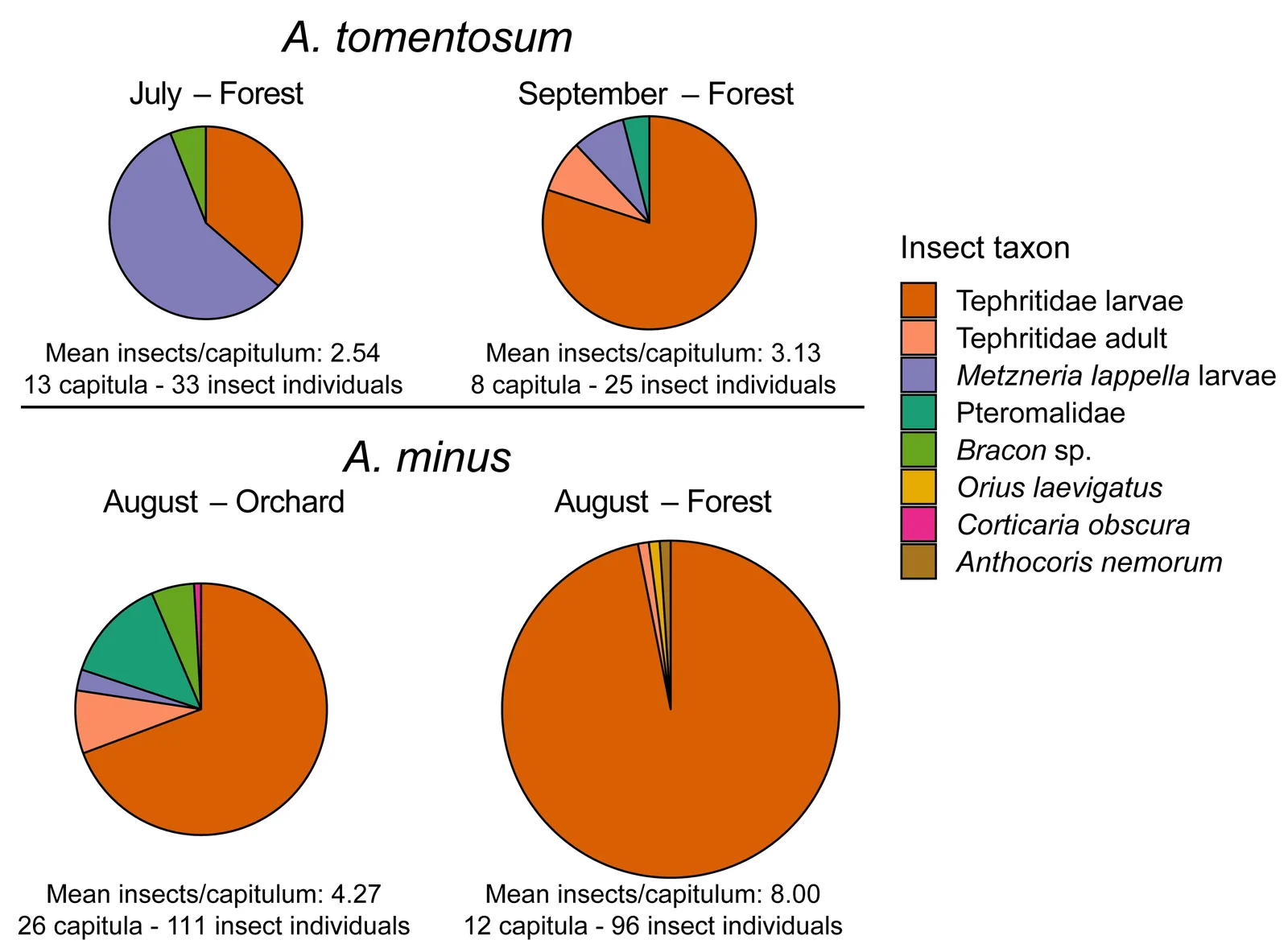
Composition and mean abundance of insects found inside *Arctium* mature capitula. The upper panel shows the insect assemblage of *A. tomentosum* in a forest habitat (OH 775), in early summer (July) and late summer (September). The lower panel shows the insect assemblage of *A. minus* in August, in an open orchard (OH 951) and a forest (OH 777). Pie chart size and the values shown below each chart indicate the mean number of insects per capitulum.

Interactor communities differed both in composition and in mean insect load per capitulum (Table S17, Fig. 5). In *A. minus*, recovered assemblage was dominated by tephritid larvae, which accounted for 69.37% of individuals in population OH 951 and 96.88% in OH 777, with mean abundances of 4.40 and eight insects per capitulum, respectively. By contrast, *A. tomentosum* showed a clear seasonal shift: during July, *M. lappella* larvae dominated the assemblage (57.57%), whereas tephritid larvae represented 36.36%, with a mean of 2.54 insects per capitulum; by September, tephritid larvae became predominant (80.00%) and *M. lappella* declined to 8%, while mean abundance rose slightly to 3.13 insects per capitulum. Tephritid adults also appeared in September, suggesting that at least some individuals may have been completing metamorphosis inside the capitula. The higher proportion of tephritid adults in OH 951 than in OH 777 population may likewise reflect environmental differences.

The most tephritid-dominated assemblages, especially in *A. minus*, coincided with lower proportions of well-formed fruits and higher predation rates than those observed in *A. tomentosum*. This was particularly evident in OH 777 population, where the almost monospecific tephritid assemblage and the highest insect load per capitulum were accompanied by significantly higher predation than in Llanars, whereas differences in well-formed and aborted fruits were not significant (Fig. S3; Table S15). By contrast, *A. tomentosum* maintained high proportions of well-formed fruits and low predation in both months despite the marked seasonal replacement from *M. lappella* to tephritids. In this context, the more heterogeneous assemblages, which also included occasional parasitoids, did not correspond to higher fruit loss, whereas the strongest reproductive damage was observed where tephritid larvae were dominant.

## Discussion

### Identity, morphological and genomic profile of the disjunct population

Morphological and nuclear data, together with genome size and repeatome composition, consistently indicate that the disjunct population corresponds to *A. tomentosum*. Based on our review of historical and contemporary distribution data, this represents the first verifiable record of the species in the Pyrenees, the Iberian Peninsula and the Balearic Islands, with the nearest known population located in the foothills of the French Alps (Fig. 2). Beyond this disjunct distribution, the cytonuclear discordance observed in the population points to a more complex evolutionary history than initially apparent. Such discordance could, in principle, arise from two non-exclusive processes: incomplete lineage sorting (ILS), where ancestral plastid variation persists across speciation events, or chloroplast capture, where past hybridization followed by backcrossing replaces most of the nuclear genome inherited from the chloroplast donor (Tsitrone, Kirkpatrick & Levin, 2003; Larson et al., 2026). Apart from the disjunct Pyrenean population, the plastid phylogeny is congruent with species-level taxonomy (Fig. 3B). This pattern is unlikely under recent ILS, which would predict random retention of ancestral plastid haplotypes across populations, and is instead more consistent with chloroplast capture (as found in annuals *Helianthus* L.; Lee-Yaw et al., 2019). In this chloroplast capture scenario, the Pyrenean population of *A. tomentosum* likely originated from a hybridization event in which *A. tomentosum* acted as the pollen donor and *A. minus* as the egg donor. This was followed by extensive backcrossing with *A. tomentosum*, restoring the morphological, nuclear genomic, and cytogenetic profile of *A. tomentosum*, with the chloroplast genome remaining as a trace of past hybridization. Taken together, these observations highlight the need to formulate a hypothesis explaining the presence of this Pyrenean population of *A. tomentosum*, which must not only account for the geographic disjunction but also for the observed pattern of chloroplast capture.

### Range contraction leading to a relict population

A possible scenario is that *A. tomentosum* was formerly widespread in the Pyrenees, or that Central European populations were once connected with this mountain range. If so, at least modest morphological or genomic differentiation from Central European material could be expected after prolonged isolation (*e.g.*, Tomasello & Oberprieler, 2017). However, all available evidence indicates a striking similarity between the Pyrenean and European individuals of *A. tomentosum*, providing no positive indication of either the age or the degree of differentiation of the Pyrenean population (Fig. 3; Fig. S1). This lack of differentiation may reflect insufficient resolution of morphological traits or molecular markers to distinguish between an ancient and a recent disjunction (*e.g.*, Vitales et al., 2019). The high degree of similarity suggests a recent colonization event, leaving insufficient time for morphological or genomic divergence from Central European source populations.

Floristic context also weighs against a long-undetected relict. Setcases, the village where the population occurs, is among the best-surveyed localities in the area (*e.g.*, Garnatje et al., 2022), making it unlikely that a truly relict population would have escaped notice for decades. In addition, reproductive-biology data at the study site indicate a local performance advantage of *A. tomentosum* over *A. minus* (Fig. 4, Fig. S3). Paradoxically, *A. tomentosum* is rare and spatially restricted whereas *A. minus*, despite its apparent disadvantage in potential recruitment, is widespread in the surroundings. This pattern is consistent with a recent arrival of *A. tomentosum*, whose persistence is currently aided by low enemy pressure rather than by long-term ecological accommodation. This interpretation is reinforced by the molecular identification of predispersal fruit predators. Tephritidae, and in particular *Tephritis bardanae*, shows host-associated genetic and morphological races linked to *A. minus* and *A. tomentosum* across Europe (Diegisser et al., 2004). Lower predation on *A. tomentosum* relative to *A. minus* at the Pyrenean site could therefore reflect incomplete local adaptation of tephritid flies to the newly available host, matching the enemy-release hypothesis, whereby recent colonists experience reduced pressure from specialist enemies (Brian & Catford, 2023). Thus, the greater predation by *M. lappella* moths on *A. tomentosum* could be explained by the availability of this resource.

Habitat context also argues against a relict explanation. The plants occur in an abandoned orchard, an anthropogenic and disturbed setting, rather than the inaccessible, long-undisturbed microhabitats that usually characterize recognized Pyrenean relicts such as *Borderea chouardii* (Gaussen) Gaussen & Heslot (García, Espadaler & Olesen, 2012). Finally, an ancient intentional introduction seems unlikely; although burdocks have recognized uses, the ethnobotanical literature of Catalonia does not mention *A. tomentosum* or any local uses of it (Rigat et al., 2007), and we found no robust records or herbarium specimens documenting its presence in the Iberian Peninsula, Balearic Islands and Pyrenees (Table S7). Taken together, these lines of evidence make a relict origin or a voluntary introduction unlikely.

### Long-distance pollination

Migratory insects can transport pollen over hundreds of kilometres, through continental regions and even during trans-oceanic flights, as documented in butterflies and other long-distance aerial migrants (Jia et al., 2022; Suchan et al., 2024). Long-distance transport of *A. tomentosum* pollen by the butterfly *Vanessa cardui* has been documented, from regions in Europe where the species grows to the Pyrenees, during the flowering period of the Pyrenean *Arctium* (Gorki et al., 2024). While long-distance pollination could, in principle, generate hybrids with *A. minus* as the maternal parent and *A. tomentosum* as the pollen donor, this scenario does not adequately explain the genetic profile of the Pyrenean population. Indeed, the observed cytonuclear discordance is only compatible with a scenario in which *A. tomentosum* acted as the pollen donor not only during the initial hybridization event but also throughout subsequent backcrossing. This would, however, be unlikely given that *A. minus* is the only parental species present in the Pyrenees.

Furthermore, in the chloroplast phylogeny, Pyrenean *A. tomentosum* does not cluster with *A. minus* from the Pyrenees (Fig. 3B), a pattern more consistent with a hybridization event occurring in the Alpine–Central European region rather than, as proposed under the long-distance pollination hypothesis, in the Pyrenees.

### Long-distance diaspore dispersal

According to this hypothesis, the Pyrenean population would have been established from diaspores transported from a more eastern population. This scenario therefore involves both chloroplast capture and long-distance dispersal. The distributions of *A. minus* and *A. tomentosum* show substantial overlap in Europe (Fig. 2), making hybridization in this region plausible. Hybrids and introgressants have indeed been documented, although gene flow between *Arctium* species appears limited due to pre and postzygotic barriers (Duistermaat, 1996; Repplinger et al., 2007). Subsequent backcrossing with *A. tomentosum* is also easily explained in this context. Long-distance diaspore dispersal is supported by the high dispersal capacity of *Arctium*, whose entire capitula with hooked involucral bracts readily attach to a wide range of animal vectors. In recent times, many *Arctium* species have expanded their distribution ranges, sometimes naturally (Javzandolgor et al., 2021). Migratory animals, livestock, and humans can transport diaspores over long distances, including inadvertent transport on clothing or equipment, thereby influencing and reshaping the species composition of localities (Bauer & Hoye, 2014). In extreme cases, hooked capitula attached to small animals, such as birds or bats, can entangle them, impairing movement and occasionally leading to death (*e.g.*, Norquay et al., 2010; Underwood & Underwood, 2013). Taken together, the available evidence supports long-distance diaspore dispersal as the most plausible explanation for the origin of the Pyrenean population of *A. tomentosum*. The occurrence of this population in an abandoned orchard could be consistent with multiple dispersal pathways, including both natural and anthropogenic vectors.

The limited sampling of *A. tomentosum* restricts our ability to assess intraspecific genomic variation and the extent of cytonuclear discordance across the species range. Consequently, it remains unclear whether the chloroplast capture inferred for the Pyrenean population represents an isolated event or a more widespread feature of certain *A. tomentosum* populations, and any inference regarding its geographic origin should therefore be considered preliminary. Nevertheless, similarities in the composition, sequence, and relative abundance of repetitive elements indicate a plausible south-central European origin rather than a north-central one (Fig. 3).

### Population integrity and dynamics

The Pyrenean population of *A. tomentosum* remains small, comprising 42 reproductive and 31 vegetative individuals, but appears to be steadily increasing since its discovery in 2021, with reproductive individuals flowering and producing fruits consistently over this period. Extensive field surveys have revealed no evidence of further hybridization between the *A. tomentosum* population and the nearby populations of *A. minus* and *A. lappa* in Setcases. Data from Central Europe, where putative hybrids are rare and introgression is limited despite sympatry (Repplinger et al., 2007), suggest that such events are unlikely under current conditions, limiting the potential impact of interspecific gene flow on population dynamics. Although the three species occupy broadly similar habitats, the *A. tomentosum* population is still small, albeit apparently expanding, indicating that niche competition resulting from its establishment, if present, is currently minimal.

Predation pressure appears lower on *A. tomentosum* than on *A. minus*, potentially reflecting its recent establishment and providing a short-term advantage to this colonizing taxon, which, although likely transient, could influence the future trajectories of the Setcases population. Biotic interaction patterns point to structured enemy pressure. Across the sampled sites, *A. minus* generally hosted a more abundant insect assemblage per capitulum than *A. tomentosum*, whereas *A. tomentosum* showed a stronger seasonal shift in assemblage composition, with late-summer samples becoming strongly dominated by tephritid larvae. Again, these patterns should be interpreted cautiously, but they are broadly compatible with previously reported trends in the genus, where herbivory on *A. minus* has been suggested to decrease toward the range margin and plant performance to increase, in line with the enemy release hypothesis (*e.g.*, Kambo & Kotanen, 2014). Likewise, habitat-related differences observed here resemble those reported in earlier studies, in which damage tended to be greater in undergrowth habitats than in open sites (Lee & Kotanen, 2017). However, expanding sampling across additional populations and multiple years would be necessary to assess whether these patterns are stable and scalable across Pyrenean populations of *A. minus*. The literature reports high predispersal losses to the same herbivores we observed; fruit predation by *M. lappella* and tephritid flies can be locally heavy (Hawthorn & Hayne, 1978; Straw, 1991) and is sensitive to plant and habitat context (Lee & Kotanen, 2015), and *Bracon* could act as enemy-of-enemy (*e.g.*, Beyarslan & Şahin, 2019). These data suggest niche partitioning among hosts, fruit predators and their parasitoids in time, space and taxonomy: tephritids partition oviposition and larval development with respect to *Arctium* phenology and localities, and the differential preference of *M. lappella* for *A. tomentosum* seen in this study. Altogether, these results support a picture in which biotic filters (structured fruit predation and parasitoid pressure, modulated by environment and phenology) shape establishment and spread after long-distance dispersal (Wu et al., 2023). Future studies could explore how variation in capitulum traits shapes differential herbivory in *A. minus* and *A. tomentosum*, while also highlighting morphological features, such as the number of involucral bracts, identified here as distinguishing the species and whose diagnostic potential should be evaluated across additional populations.

## Conclusions

The isolated Pyrenean population of *A. tomentosum* is best explained as a recent long-distance colonist rather than the result of a long-distance pollination or an undetected relict. Morphological and genomic affinities to Central European *A. tomentosum* suggest an origin in or near this region. Patterns of fruit predation may be compatible with enemy release on *A. tomentosum*, suggesting that structured biotic filters could modulate establishment and spread after arrival. This system offers a valuable opportunity to understand how disjunctions arise and how newly established populations integrate into local ecological networks.

##  Supplemental Information

10.7717/peerj.21692/supp-1 Supplemental Information 1 Herbarium voucher information and morphological measurements and assignments of quantitative and qualitative traits Qualitative traits were coded following the order in which they appear in the table. Inner-bract apex shape was treated as an ordinal variable and coded as 0 = acute, 1 = cuspidate, and 2 = truncate. Involucrum pubescence was also treated as ordinal and coded as 0 = glabrous, 1 = sparse, and 2 = dense. Corolla indumentum, here corresponding to glandular hairs, was coded as an ordinal trait with 0 = glabrous, 1 = glabrescent, and 2 = abundant. Synflorescence architecture was treated as a nominal variable and coded as an unordered factor. Flower position relative to the involucral bracts was treated as a binary variable, with 0 = flowers remaining below the bracts and 1 = flowers extending beyond them.

10.7717/peerj.21692/supp-2 Supplemental Information 2 Genome size data for *Arctium* accessions in Pyrenees

10.7717/peerj.21692/supp-3 Supplemental Information 3 Genome size data for *Arctium* accessions in Pyrenees

10.7717/peerj.21692/supp-4 Supplemental Information 4 Raw data at capitulum level for the reproductive success analysis For each capitulum, we recorded the total number of flowers, total number of fruits, and the number of well-formed, aborted, and predated fruits. The column Rest indicates the difference between the total number of flowers and the total number of fruits.

10.7717/peerj.21692/supp-5 Supplemental Information 5 Raw data at capitulum level used for the functional trait analysis For each capitulum, we recorded capitulum height, involucre diameter, involucre height, number of involucral bracts, and number of flowers. Measurements of the first three trait variables are given in millimetres (mm).

10.7717/peerj.21692/supp-6 Supplemental Information 6 Voucher information and morphogroup composition of the insect specimens included in the analyses Shaded cells indicate morphogroups for which one specimen was selected for molecular identification. When multiple individuals of the same morphogroup were available, the specimen chosen was the one that was intact and showed the best external condition for extraction.

10.7717/peerj.21692/supp-7 Supplemental Information 7 GBIF records of *Arctium tomentum* from Pyrenees dismissed from the dataset Region: Iberian Peninsula (IB), Balearic Islands (BA), Pyrenees (P).

10.7717/peerj.21692/supp-8 Supplemental Information 8 Comparative analysis of repetitive elements in *Arctium* accessions Geographic origin: Pyrenees (P), Slovenian Alps (SA), Alps (A).

10.7717/peerj.21692/supp-9 Supplemental Information 9 Pairwise R^2^ values from linear regressions comparing estimated repeat cluster copy numbers across individuals Sample provenance: Pyrenees (P), Alps (A), Slovenian Alps (SA) (see Table S3). Data derived from Table S8.

10.7717/peerj.21692/supp-10 Supplemental Information 10 Mean differences in functional traits between *Arctium minus* and *A. tomentosum* Mean is based on 27 terminal fresh capitula for *A. minus* and on 25 for *A. tomentosum*. Significant *p*-values are highlighted in bold.

10.7717/peerj.21692/supp-11 Supplemental Information 11 Variance partitioning for the random-effects structure across fruit categories Analyses were performed under two alternative assumptions: excluding the discrepancy between flowers and fruits, indicated in the Category column as “without”, or treating this discrepancy as fully predated fruits, indicated as “with”.

10.7717/peerj.21692/supp-12 Supplemental Information 12 Model fit comparison and diagnostic statistics for GLMMs fitted to well-formed, aborted, and predated fruits For the last variable, analyses were performed under two alternative assumptions: excluding the discrepancy between flowers and fruits, indicated in the Model column as “without”, or treating this discrepancy as fully predated fruits, indicated as “with”. Significant *p*-values are highlighted in bold.

10.7717/peerj.21692/supp-13 Supplemental Information 13 Fixed-effect estimates from GLMMs for well-formed, predated and aborted fruits Species was included as a fixed effect, with *A. minus* used as the reference level. Analyses were performed under two alternative assumptions: excluding the discrepancy between flowers and fruits, indicated in the Model column as “without”, or treating this discrepancy as fully predated fruits, indicated as “with”. Significant *p*-values are highlighted in bold.

10.7717/peerj.21692/supp-14 Supplemental Information 14 Empirical and model-based differences in lower-to-fruit rates between *Arctium minus* and *A. tomentosum* Analyses were performed under two alternative assumptions: excluding the discrepancy between flowers and fruits, indicated in the Category column as “without”, or treating this discrepancy as fully predated fruits, indicated as “with”. Significant *p*-values are highlighted in bold.

10.7717/peerj.21692/supp-15 Supplemental Information 15 Non-parametric pairwise comparisons of conversion rates in *A. minus* and *A. tomentosum* The multiple comparison based on the Kruskal–Wallis test for August 2022 among the OH 728, OH 775 and OH777 *A. minus* populations was omitted, as the mean and standard deviation for each population in that month and year are already provided, and the test was not significant. Significant *p*-values are highlighted in bold. Analyses were performed under two alternative assumptions in predated Response column: excluding the discrepancy between flowers and fruits, indicated in the Coding column as “without”, or treating this discrepancy as fully predated fruits, indicated as “with.

10.7717/peerj.21692/supp-16 Supplemental Information 16 inear models relating flower production to well-formed fruits and predation to abortion relative to well-formed fruits under both coding schemes Significant *p*-values are highlighted in bold.

10.7717/peerj.21692/supp-17 Supplemental Information 17 Composition in percentage of insect assemblages per population and month for *A. minus* and *A. tomentosum* Data derived from Table S6.

10.7717/peerj.21692/supp-18 Supplemental Information 18 cox1 sequences for barcoding The art codes link each sequence to the one referenced in table S6

10.7717/peerj.21692/supp-19 Supplemental Information 19 NeighborNet network based on morphological traits scored from herbarium specimens Green, blue, and purple outlines group specimens of *A. lappa*, *A. minus*, and *A. tomentosum*, respectively. Individuals identified as hybrids were also incorporated: *Arctium*×*ambiguum* (Čelak.) Nyman, *Arctium*×*mixtum* (Simonk.) Nyman and* Arctium* × *nothum* (Ruhmer) Hallier (corresponding to hybrids between *A. lappa* × *A. tomentosum*, *A. minus*×*A. tomentosum*, and *A. lappa*×* A. minus*, respectively; POWO, 2026). Letters following each taxon name indicate the country of origin of the herbarium specimen, whereas numbers identify the corresponding individual in Supplementary Table S1. The disjunct Pyrenean population, indicated in the network by the straight purple line, corresponds to *A. tomentosum* ES 17.

10.7717/peerj.21692/supp-20 Supplemental Information 20 Flow histogram obtained from the combined analysis of A. tomentosum (peak 1), A. lappa (peak 2), A. minus (peak 3) and the calibration standard P. sativum (9.09 pg/2C, peak 4)

10.7717/peerj.21692/supp-21 Supplemental Information 21 Comparison schemes used to assess temporal and spatial variation in fruit conversion rates The first row shows broad interspecific and interannual comparisons, including differences between *A. minus* and *A. tomentosum* within each year and between consecutive years within each species, with data pooled across populations and sampling months (ns = non significant; **,* p* ¡ 0.01; ***, *p* ¡ 0.001; Wilcoxon rank-sum test). The second row examines spatial variation in *A. minus* within a single year, comparing all three populations together and each population pair separately. The third row presents finer-scale contrasts: monthly variation within *A. tomentosum* in 2023, interannual differences within one *A. minus* population, and spatial differences between two *A. minus* populations sampled in the same month. See Supplementary Table S15 for details. The x-axis shows the fruit conversion categories: WF, conversion rate from flowers to well-formed fruits; AF, conversion rate to aborted fruits; and PF, conversion rate to predated fruits under the two coding schemes. PF yes includes the difference between flower number and recovered fruits as fully predated fruits, whereas PF no considers only the directly counted predated fruits.

## Additional Information and Declarations

### Data Availability

The following information was supplied regarding data availability: The raw data is available in the Supplemental Files. The SRA *Arctium* data are available at GenBank: - BioProject: PRJNA1450918.
