# Supplementary material for: Evaluating three alternative scenarios for the origin of a disjunct Arctium tomentosum population in the Pyrenees
Source: PeerJ. 2026 Sep 7;14:e21692. doi: 10.7717/peerj.21692 (PMC13564400; doi:10.7717/peerj.21692)
Supplement: Supplementary file 1 [file peerj-14-21692-s020.pdf]

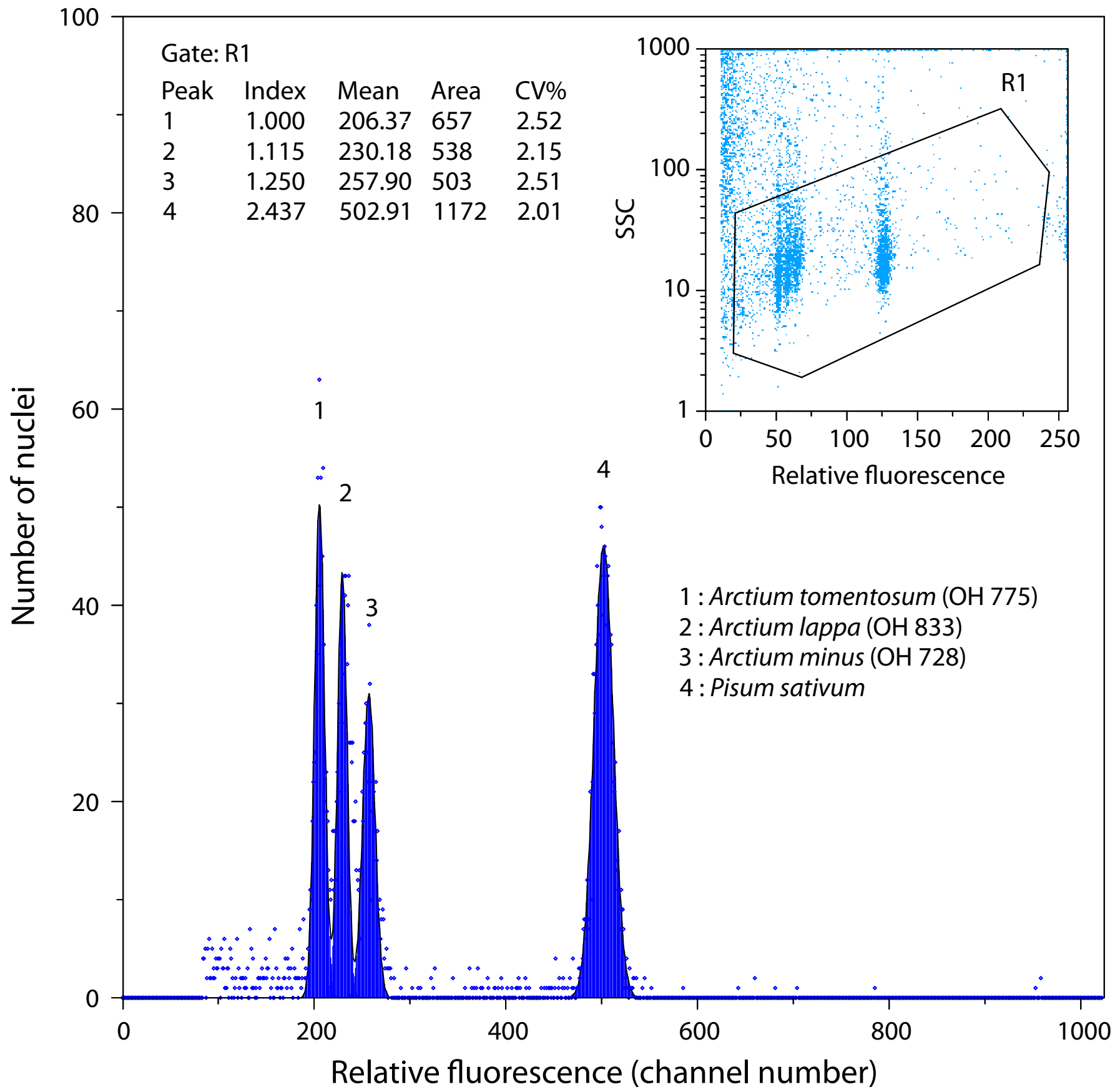
Gate: R1
Peak Index Mean Area CV%
1 1.000 206.37 657 2.52
2 1.115 230.18 538 2.15
3 1.250 257.90 503 2.51
4 2.437 502.91 1172 2.01
Number of nuclei
Relative fluorescence (channel number)
1 : Arctium tomentosum (OH 775)
2 : Arctium lappa (OH 833)
3 : Arctium minus (OH 728)
4 : Pisum sativum
SSC
Relative fluorescence
R1
