# Supplementary material for: Evaluating three alternative scenarios for the origin of a disjunct Arctium tomentosum population in the Pyrenees
Source: PeerJ. 2026 Sep 7;14:e21692. doi: 10.7717/peerj.21692 (PMC13564400; doi:10.7717/peerj.21692)
Supplement: Supplementary file 2 [file peerj-14-21692-s021.pdf]

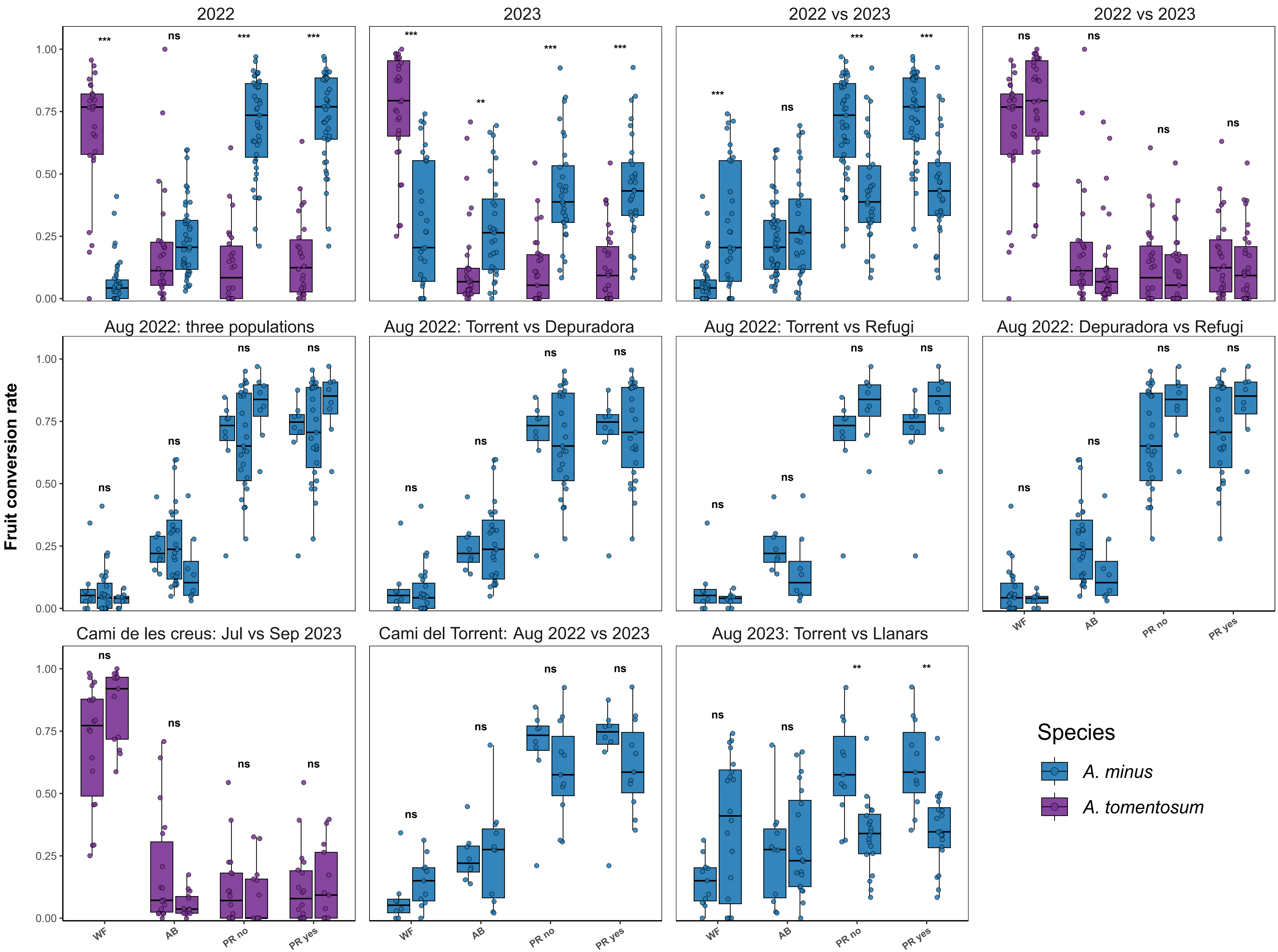

2022
2023
2022 vs 2023
2022 vs 2023
Aug 2022: three populations
Aug 2022: Torrent vs Depuradora
Aug 2022: Torrent vs Refugi
Aug 2022: Depuradora vs Refugi
Cami de les creus: Jul vs Sep 2023
Cami del Torrent: Aug 2022 vs 2023
Aug 2023: Torrent vs Llanars
Fruit conversion rate
WF
AB
PR no
PR yes
Species
A. minus
A. tomentosum
