# Supplementary material for: Evaluating three alternative scenarios for the origin of a disjunct Arctium tomentosum population in the Pyrenees
Source: PeerJ. 2026 Sep 7;14:e21692. doi: 10.7717/peerj.21692 (PMC13564400; doi:10.7717/peerj.21692)
Supplement: Supplementary file 3 [file peerj-14-21692-s001.docx]

**Supplemental Table 1.** Herbarium voucher information and morphological measurements and assignments of quantitative and qualitative traits. Qualitative traits were coded following the order in which they appear in the table. Inner-bract apex shape was treated as an ordinal variable and coded as 0 = acute, 1 = cuspidate, and 2 = truncate. Involucrum pubescence was also treated as ordinal and coded as 0 = glabrous, 1 = sparse, and 2 = dense. Corolla indumentum, here corresponding to glandular hairs, was coded as an ordinal trait with 0 = glabrous, 1 = glabrescent, and 2 = abundant. Synflorescence architecture was treated as a nominal variable and coded as an unordered factor. Flower position relative to the involucral bracts was treated as a binary variable, with 0 = flowers remaining below the bracts and 1 = flowers extending beyond them.

| **Accession data** | | | | | | **Quantitative traits (mm)** | | | | | | | | | **Qualitative traits** | | | | |
| --- | --- | --- | --- | --- | --- | --- | --- | --- | --- | --- | --- | --- | --- | --- | --- | --- | --- | --- | --- |
| **Species** | **Code^1^** | **Herbarium** | **Voucher** | **Country^2^** |  | **Peduncle length** | **Involucre diameter** | **Involucre height** | **Involucre diameter without apex^3^** | **Involucre height without apex^3^** | **Middle bract length** | **Middle bract width** | **Inner bract length** | **Inner bract width** | **Inner bract apex** | **Involucre pubescense** | **Corolla indumentum** | **Synflorescence architecture** | **Flowers over involucre** |
| *Arctium lappa* | 1 | BC | 9637795 | UK |  | 33.44 | 34 | 39.28 | 21.03 | 22.05 | 16.69 | 1.75 | 18.32 | 0.97 | acute | glabrous | glabrous | corymbiform | no |
| *Arctium lappa* | 2 | JACA | R311691 | FR |  | 34.78 | 31.94 | 28.15 | 20.1 | 16.81 | 14.97 | 1.63 | 15.81 | 1.16 | acute | glabrous | glabrous | corymbiform | no |
| *Arctium lappa* | 3 | BC | 133126 | FR |  | 34.25 | 37 | 30.48 | 21.26 | 12.61 | 13.18 | 1.16 | 13.64 | 1.06 | acute | glabrous | glabrous | corymbiform | no |
| *Arctium lappa* | 4 | BC | 630082 | FR |  | 42.3 | 36.1 | 33.37 | 19.77 | 17.84 | 12.89 | 0.95 | 15.44 | 1.75 | acute | glabrous | glabrous | corymbiform | no |
| *Arctium lappa* | 5 | BC | OH833 | ES |  | 75.41 | 38.48 | 31.84 | 25.41 | 12.2 | 14.7 | 1.07 | 18.03 | 1.19 | acute | glabrous | abundant | corymbiform | no |
| *Arctium minus* | 6 | JACA | R178677 | ES |  | 15.21 | 29.21 | 20.66 | 19.05 | 9.77 | 11.77 | 1.01 | 12.33 | 0.62 | acute | glabrous | glabrous | spiciform | yes |
| *Arctium minus* | 7 | JACA | R274488 | ES |  | 13.67 | 28.55 | 13.8 | 19.68 | 11.89 | 11.1 | 0.92 | 11.73 | 0.62 | acute | glabrous | glabrous | spiciform | yes |
| *Arctium minus* | 8 | JACA | R87906 | ES |  | 12.14 | 23.24 | 14.63 | 14.61 | 9.09 | 11.38 | 0.84 | 9.05 | 0.66 | acute | glabrous | glabrescent | spiciform | yes |
| *Arctium minus* | 9 | BC | BC-688043 | ES |  | 0 | 23.73 | 17.24 | 15 | 13.9 | 8.18 | 0.95 | 11.09 | 0.9 | acute | glabrous | glabrous | spiciform | yes |
| *Arctium minus* | 10 | BC | 110164 | ES |  | 5.35 | 26.86 | 20.99 | 15.1 | 11.95 | 11.55 | 1 | 11.7 | 1.17 | acute | glabrous | glabrous | spiciform | yes |
| *Arctium minus* | 11 | JACA | R453784 | NL |  | 10.26 | 21.89 | 12.16 | 17.21 | 9.75 | 6.93 | 1.22 | 11.85 | 0.81 | acute | sparse | glabrescent | spiciform | yes |
| *Arctium minus* | 12 | BC | OH728 | ES |  | 6.19 | 19.29 | 15.16 | 11.2 | 7.58 | 9.99 | 0.87 | 9.07 | 1.21 | acute | glabrous | glabrous | spiciform | yes |
| *Arctium tomentosum* | 13 | JACA | R311689 | CZ |  | 28 | 25.62 | 16.68 | 18.41 | 12.19 | 9.61 | 1.32 | 12.83 | 1.41 | truncate | dense | abundant | corymbiform | no |
| *Arctium tomentosum* | 14 | JACA | R311690 | CZ |  | 23.6 | 26.11 | 20.15 | 19.94 | 17.36 | 10.15 | 1.16 | 11.45 | 1.18 | truncate | dense | abundant | corymbiform | yes |
| *Arctium tomentosum* | 15 | JACA | 33014 | CZ |  | 18.23 | 26.06 | 24.87 | 18.95 | 18.04 | 10.29 | 0.96 | 14.46 | 2.01 | truncate | dense | abundant | corymbiform | yes |
| *Arctium tomentosum* | 16 | JACA | R273389 | FR |  | 14.96 | 20.43 | 19.46 | 14 | 11.09 | 9.78 | 1.06 | 13.2 | 1.64 | cuspidate | dense | abundant | corymbiform | no |
| *Arctium tomentosum* | 17 | BC | OH775 | ES |  | 27.32 | 21.08 | 14.82 | 11.86 | 9.61 | 8.78 | 1.11 | 11.75 | 1.9 | truncate | dense | abundant | corymbiform | yes |
| *Arctium x ambiguum* | 18 | BC | 877982 | AT |  | 40.53 | 28.24 | 23.08 | 15.48 | 13.43 | 11.47 | 1.16 | 16.81 | 1.91 | acute | sparse | glabrous | corymbiform | yes |
| *Arctium x ambiguum* | 19 | JACA | R258690 | FI |  | 23.24 | 29.49 | 27.26 | 22.16 | 19.49 | 11.59 | 1.64 | 14.27 | 1.89 | truncate | sparse | abundant | corymbiform | yes |
| *Arctium x ambiguum* | 20 | BC | 33027 | ND |  | 59.86 | 35.41 | 26.95 | 22.79 | 13.03 | 12.76 | 0.99 | 16.4 | 1.35 | acute | sparse | glabrescent | corymbiform | no |
| *Arctium x mixtum* | 21 | BC | BC-987107 | AT |  | 10.74 | 22.2 | 20.76 | 12.34 | 11.79 | 12.44 | 0.89 | 14.46 | 0.63 | acute | sparse | glabrous | subcorymbiform | yes |
| *Arctium x tothum* | 22 | JACA | JACA R63786 | NL |  | 19.62 | 23.21 | 17.04 | 16.8 | 11.31 | 11.55 | 1.14 | 15.6 | 1.24 | acute | glabrous | glabrescent | subcorymbiform | yes |

^1^Code number: identifies each herbarium specimen in the network (Figure S1). Country: UK (United Kingdom). FR (France), ES (Spain), NL (Netherlands), CZ (Czech Republic), FI (Finland), ND (No data), AT (Austria). ^3^Measurements of involucre width and height were taken both including and excluding the patent portion of the involucral bracts, following *Duistermaat (1996)*.
