# Supplementary material for: Evaluating three alternative scenarios for the origin of a disjunct Arctium tomentosum population in the Pyrenees
Source: PeerJ. 2026 Sep 7;14:e21692. doi: 10.7717/peerj.21692 (PMC13564400; doi:10.7717/peerj.21692)
Supplement: Supplementary file 4 [file peerj-14-21692-s002.docx]

**Supplemental Table 2**. Genome size data for *Arctium* accessions in Pyrenees.

| ***Arctium* species** | **Pop** | **GPS** | **2C (pg) ± SD^1^** | **CV_plt_^2^** | **CV_std_^3^** |
| --- | --- | --- | --- | --- | --- |
| *A. lappa* | OH 833 | 42°22'24"N 2°17'55"E | 4.17 ± 0.02 | 3.09 | 2.08 |
| *A. minus* | OH 779 | 42°23'27"N 2°17'40"E | 4.67 ± 0.05 | 2.82 | 2.49 |
| *A. minus* | OH 728 | 42°22'20"N 2°17'58"E | 4.68 ± 0.05 | 3.15 | 2.89 |
| *A.* *tomentosum* | OH 775 | 42°22'29"N 2°17'59"E | 3.89 ± 0.02 | 3.00 | 2.77 |
| ^1^SD: standard deviation. ^2^CV_plt_: coefficient of variation for *Arctium* accessions (in %). ^3^CV_std_: coefficient of variation for the calibration standard (in %). | | | | | |
