# Supplementary material for: Evaluating three alternative scenarios for the origin of a disjunct Arctium tomentosum population in the Pyrenees
Source: PeerJ. 2026 Sep 7;14:e21692. doi: 10.7717/peerj.21692 (PMC13564400; doi:10.7717/peerj.21692)
Supplement: Supplementary file 5 [file peerj-14-21692-s003.docx]

**Supplemental Table 3.** Genomic vouchers and accession information for *Arctium* and *Cousinia* samples used in the repeatome and phylogenetic analyses.

| **Species** | **Geographic acronym^1^** | **Voucher** | **Country^2^** | **Type of data** | **NCBI Code** |
| --- | --- | --- | --- | --- | --- |
| *Arctium lappa* | P | OH 833 | ES | Genome skimming | SRR38014131 |
| *Arctium lappa* | A | GR000466 | FR | Genome skimming | ERR14012663 |
| *Arctium minus* | P | OH 728 | ES | Genome skimming | SRR38014132 |
| *Arctium minus* | A | GR004440 | CH | Genome skimming | ERR14012261 |
| *Arctium tomentosum* | P | OH 775 | ES | Genome skimming | SRR38014133 |
| *Arctium tomentosum* | SA | OH 476 | SI | Genome skimming | SRR38014130 |
| *Arctium tomentosum* | A | GR003695 | CH | Genome skimming | ERR14046923 |
| *Arctium nemorosum* | A | GR000802 | FR | Genome skimming | ERR14029927 |
| *Cousinia proxima*^3^ | - | - | - | Complete chloroplast | PQ240605.1 |
| *Cousinia gomolitzkii*^4^ | - | - | - | ITS and LSU rDNA | PP109402.1 |

^1^Abbreviations used to indicate geographic origin, distinguishing between the data generated in this study and those obtained from *Lavergne et al. (2025)*. *Arctium* sequences from the Slovenian Alps (SA) and the Pyrenees (P) were generated in this study, whereas those from *Lavergne et al. (2025)* correspond to the remaining Alpine samples (A). ^2^Country abbreviations for sample origin are as follows: ES (Spain), FR (France), CH (Switzerland), SI (Slovenia). ³Taxon used to root the chloroplast tree; sequence from *Karimov et al. (2025)*. ⁴Taxon used to root the nuclear tree; sequence from *Kenesbay et al. (2025)*.
