# Supplementary material for: Evaluating three alternative scenarios for the origin of a disjunct Arctium tomentosum population in the Pyrenees
Source: PeerJ. 2026 Sep 7;14:e21692. doi: 10.7717/peerj.21692 (PMC13564400; doi:10.7717/peerj.21692)
Supplement: Supplementary file 6 [file peerj-14-21692-s006.docx]

**Supplemental Table 6.** Voucher information and morphogroup composition of the insect specimens included in the analyses. Shaded cells indicate morphogroups for which one specimen was selected for molecular identification. When multiple individuals of the same morphogroup were available, the specimen chosen was the one that was intact and showed the best external condition for extraction.

| **Voucher information** | | | | | **Morphogroup** | | | | | | | | |
| --- | --- | --- | --- | --- | --- | --- | --- | --- | --- | --- | --- | --- | --- |
|  |  |  |  |  | **Diptera** | | | **Hemiptera** | | **Lepidoptera** | **Hymenoptera parasitoid** | | **Coleoptera** |
| **Taxon** | **Pop.** | **GPS** | **Date** | **Molecular Identification**  **(NCBI code)** | **Larvae**  **1** | **Larvae**  **2** | **Adult**  **1** | **Adult**  **1** | **Adult**  **2** | **Larvae**  **1** | **Adult**  **1** | **Adult**  **2** | **Adult**  **1** |
| *A. tomentosum* | OH 775 | 42°22'29"N 2°17'59"E | 6/9/2023 | *Tephritis bardanae* (PZ322886;art5) | 0 | 0 | 1 | 0 | 0 | 0 | 0 | 0 | 0 |
| *A. tomentosum* | OH 775 | 42°22'29"N 2°17'59"E | 6/9/2023 |  | 5 | 0 | 0 | 0 | 0 | 0 | 0 | 0 | 0 |
| *A. tomentosum* | OH 775 | 42°22'29"N 2°17'59"E | 6/9/2023 |  | 3 | 0 | 0 | 0 | 0 | 0 | 0 | 0 | 0 |
| *A. tomentosum* | OH 775 | 42°22'29"N 2°17'59"E | 6/9/2023 | *Terellia tussilaginis* (PZ322883;art6)  *Metzneria lappella* (PZ322878;art4) | 2 | 0 | 0 | 0 | 0 | 1 | 0 | 0 | 0 |
| *A. tomentosum* | OH 775 | 42°22'29"N 2°17'59"E | 6/9/2023 |  | 6 | 0 | 0 | 0 | 0 | 0 | 0 | 0 | 0 |
| *A. tomentosum* | OH 775 | 42°22'29"N 2°17'59"E | 6/9/2023 | *Wolbachia* sp. (PZ673059; art7) | 2 | 0 | 0 | 0 | 0 | 0 | 1 | 0 | 0 |
| *A. tomentosum* | OH 775 | 42°22'29"N 2°17'59"E | 6/9/2023 |  | 2 | 0 | 1 | 0 | 0 | 0 | 0 | 0 | 0 |
| *A. tomentosum* | OH 775 | 42°22'29"N 2°17'59"E | 6/9/2023 |  | 0 | 0 | 0 | 0 | 0 | 1 | 0 | 0 | 0 |
| *A. tomentosum* | OH 775 | 42°22'29"N 2°17'59"E | 24/7/2023 |  | 0 | 0 | 0 | 0 | 0 | 2 | 0 | 0 | 0 |
| *A. tomentosum* | OH 775 | 42°22'29"N 2°17'59"E | 24/7/2023 |  | 0 | 0 | 0 | 0 | 0 | 2 | 0 | 0 | 0 |
| *A. tomentosum* | OH 775 | 42°22'29"N 2°17'59"E | 24/7/2023 |  | 0 | 0 | 0 | 0 | 0 | 3 | 0 | 0 | 0 |
| *A. tomentosum* | OH 775 | 42°22'29"N 2°17'59"E | 24/7/2023 |  | 0 | 0 | 0 | 0 | 0 | 1 | 0 | 0 | 0 |
| *A. tomentosum* | OH 775 | 42°22'29"N 2°17'59"E | 24/7/2023 | *Metzneria lappella* (PZ322879; art1) | 0 | 0 | 0 | 0 | 0 | 2 | 0 | 0 | 0 |
| *A. tomentosum* | OH 775 | 42°22'29"N 2°17'59"E | 24/7/2023 |  | 0 | 0 | 0 | 0 | 0 | 3 | 0 | 0 | 0 |
| *A. tomentosum* | OH 775 | 42°22'29"N 2°17'59"E | 24/7/2023 |  | 0 | 0 | 0 | 0 | 0 | 1 | 0 | 0 | 0 |
| *A. tomentosum* | OH 775 | 42°22'29"N 2°17'59"E | 24/7/2023 |  | 3 | 0 | 0 | 0 | 0 | 0 | 0 | 0 | 0 |
| *A. tomentosum* | OH 775 | 42°22'29"N 2°17'59"E | 24/7/2023 |  | 2 | 0 | 0 | 0 | 0 | 0 | 0 | 0 | 0 |
| *A. tomentosum* | OH 775 | 42°22'29"N 2°17'59"E | 24/7/2023 | *Terellia tussilaginis* (PZ322881; art2)  *Bracon* sp. (PZ322872; *art3*) | 7 | 0 | 0 | 0 | 0 | 2 | 0 | 2 | 0 |
| *A. tomentosum* | OH 775 | 42°22'29"N 2°17'59"E | 27/7/2023 |  | 0 | 0 | 0 | 0 | 0 | 1 | 0 | 0 | 0 |
| *A. tomentosum* | OH 775 | 42°22'29"N 2°17'59"E | 27/7/2023 |  | 0 | 0 | 0 | 0 | 0 | 1 | 0 | 0 | 0 |
| *A. tomentosum* | OH 775 | 42°22'29"N 2°17'59"E | 27/7/2023 |  | 0 | 0 | 0 | 0 | 0 | 1 | 0 | 0 | 0 |
| *A. minus* | OH 777 | 42°22'37"N 2°18'24"E | 7/8/2023 |  | 6 | 0 | 0 | 0 | 0 | 0 | 0 | 0 | 0 |
| *A. minus* | OH 777 | 42°22'37"N 2°18'24"E | 7/8/2023 | *Tephritis bardanae* (PZ322887; art8) | 8 | 0 | 1 | 0 | 0 | 0 | 0 | 0 | 0 |
| *A. minus* | OH 777 | 42°22'37"N 2°18'24"E | 7/8/2023 | *Terellia tussilaginis* (PZ322884;art9) | 19 | 0 | 0 | 0 | 0 | 0 | 0 | 0 | 0 |
| *A. minus* | OH 777 | 42°22'37"N 2°18'24"E | 7/8/2023 |  | 2 | 0 | 0 | 0 | 0 | 0 | 0 | 0 | 0 |
| *A. minus* | OH 777 | 42°22'37"N 2°18'24"E | 7/8/2023 |  | 3 | 0 | 0 | 0 | 0 | 0 | 0 | 0 | 0 |
| *A. minus* | OH 777 | 42°22'37"N 2°18'24"E | 7/8/2023 | *Terellia tussilaginis* (PZ322882;art10)  *Orius laevigatus* (PZ322876;art12) | 0 | 1 | 0 | 1 | 0 | 0 | 0 | 0 | 0 |
| *A. minus* | OH 777 | 42°22'37"N 2°18'24"E | 7/8/2023 | *Anthocoris nemorum* (PZ322875;art11) | 9 | 0 | 0 | 0 | 1 | 0 | 0 | 0 | 0 |
| *A. minus* | OH 777 | 42°22'37"N 2°18'24"E | 7/8/2023 |  | 15 | 0 | 0 | 0 | 0 | 0 | 0 | 0 | 0 |
| *A. minus* | OH 777 | 42°22'37"N 2°18'24"E | 7/8/2023 |  | 3 | 0 | 0 | 0 | 0 | 0 | 0 | 0 | 0 |
| *A. minus* | OH 777 | 42°22'37"N 2°18'24"E | 7/8/2023 |  | 4 | 0 | 0 | 0 | 0 | 0 | 0 | 0 | 0 |
| *A. minus* | OH 777 | 42°22'37"N 2°18'24"E | 7/8/2023 |  | 5 | 0 | 0 | 0 | 0 | 0 | 0 | 0 | 0 |
| *A. minus* | OH 777 | 42°22'37"N 2°18'24"E | 7/8/2023 |  | 18 | 0 | 0 | 0 | 0 | 0 | 0 | 0 | 0 |
| *A. minus* | OH 951 | 42°18'30.0"N 2°20'14.9"E | 17/8/2023 |  | 1 | 0 | 1 | 0 | 0 | 0 | 0 | 0 | 0 |
| *A. minus* | OH 951 | 42°18'30.0"N 2°20'14.9"E | 17/8/2023 |  | 4 | 0 | 0 | 0 | 0 | 0 | 0 | 0 | 0 |
| *A. minus* | OH 951 | 42°18'30.0"N 2°20'14.9"E | 17/8/2023 | *Tephritis bardanae* (PZ322888;art15) | 2 | 0 | 3 | 0 | 0 | 0 | 1 | 0 | 0 |
| *A. minus* | OH 951 | 42°18'30.0"N 2°20'14.9"E | 17/8/2023 | *Terellia tussilaginis* (PZ322885; art16) | 5 | 0 | 0 | 0 | 0 | 0 | 0 | 0 | 0 |
| *A. minus* | OH 951 | 42°18'30.0"N 2°20'14.9"E | 17/8/2023 |  | 1 | 0 | 2 | 0 | 0 | 0 | 0 | 0 | 0 |
| *A. minus* | OH 951 | 42°18'30.0"N 2°20'14.9"E | 17/8/2023 |  | 0 | 0 | 0 | 0 | 0 | 0 | 1 | 0 | 0 |
| *A. minus* | OH 951 | 42°18'30.0"N 2°20'14.9"E | 17/8/2023 |  | 2 | 0 | 0 | 0 | 0 | 0 | 0 | 0 | 0 |
| *A. minus* | OH 951 | 42°18'30.0"N 2°20'14.9"E | 17/8/2023 |  | 6 | 0 | 0 | 0 | 0 | 0 | 0 | 0 | 0 |
| *A. minus* | OH 951 | 42°18'30.0"N 2°20'14.9"E | 17/8/2023 |  | 0 | 0 | 1 | 0 | 0 | 0 | 0 | 0 | 0 |
| *A. minus* | OH 951 | 42°18'30.0"N 2°20'14.9"E | 17/8/2023 |  | 0 | 0 | 1 | 0 | 0 | 0 | 0 | 0 | 0 |
| *A. minus* | OH 951 | 42°18'30.0"N 2°20'14.9"E | 17/8/2023 |  | 2 | 0 | 0 | 0 | 0 | 0 | 0 | 0 | 0 |
| *A. minus* | OH 951 | 42°18'30.0"N 2°20'14.9"E | 17/8/2023 |  | 4 | 0 | 0 | 0 | 0 | 0 | 1 | 0 | 0 |
| *A. minus* | OH 951 | 42°18'30.0"N 2°20'14.9"E | 17/8/2023 |  | 2 | 0 | 0 | 0 | 0 | 0 | 4 | 0 | 0 |
| *A. minus* | OH 951 | 42°18'30.0"N 2°20'14.9"E | 17/8/2023 | *Metzneria lappella* (PZ322880;art14) *Wolbachia* sp. (PZ673060;art17) | 0 | 0 | 0 | 0 | 0 | 2 | 4 | 0 | 0 |
| *A. minus* | OH 951 | 42°18'30.0"N 2°20'14.9"E | 17/8/2023 | *Corticaria* *obscura* (PZ322877;art13) | 1 | 0 | 0 | 0 | 0 | 0 | 0 | 0 | 1 |
| *A. minus* | OH 951 | 42°18'30.0"N 2°20'14.9"E | 17/8/2023 |  | 2 | 0 | 0 | 0 | 0 | 1 | 0 | 1 | 0 |
| *A. minus* | OH 951 | 42°18'30.0"N 2°20'14.9"E | 17/8/2023 | *Bracon* sp. (PZ322874;art19) | 2 | 0 | 0 | 0 | 0 | 0 | 0 | 1 | 0 |
| *A. minus* | OH 951 | 42°18'30.0"N 2°20'14.9"E | 17/8/2023 | *Bracon* sp. (PZ322873;art18) | 2 | 0 | 0 | 0 | 0 | 0 | 0 | 4 | 0 |
| *A. minus* | OH 951 | 42°18'30.0"N 2°20'14.9"E | 17/8/2023 |  | 7 | 0 | 0 | 0 | 0 | 0 | 0 | 0 | 0 |
| *A. minus* | OH 951 | 42°18'30.0"N 2°20'14.9"E | 17/8/2023 |  | 4 | 0 | 0 | 0 | 0 | 0 | 4 | 0 | 0 |
| *A. minus* | OH 951 | 42°18'30.0"N 2°20'14.9"E | 17/8/2023 |  | 2 | 0 | 1 | 0 | 0 | 0 | 0 | 0 | 0 |
| *A. minus* | OH 951 | 42°18'30.0"N 2°20'14.9"E | 17/8/2023 |  | 2 | 0 | 0 | 0 | 0 | 0 | 0 | 0 | 0 |
| *A. minus* | OH 951 | 42°18'30.0"N 2°20'14.9"E | 17/8/2023 |  | 7 | 0 | 0 | 0 | 0 | 0 | 0 | 0 | 0 |
| *A. minus* | OH 951 | 42°18'30.0"N 2°20'14.9"E | 17/8/2023 |  | 4 | 0 | 0 | 0 | 0 | 0 | 0 | 0 | 0 |
| *A. minus* | OH 951 | 42°18'30.0"N 2°20'14.9"E | 17/8/2023 |  | 6 | 0 | 0 | 0 | 0 | 0 | 0 | 0 | 0 |
| *A. minus* | OH 951 | 42°18'30.0"N 2°20'14.9"E | 17/8/2023 |  | 9 | 0 | 0 | 0 | 0 | 0 | 0 | 0 | 0 |
