# Supplementary material for: Evaluating three alternative scenarios for the origin of a disjunct Arctium tomentosum population in the Pyrenees
Source: PeerJ. 2026 Sep 7;14:e21692. doi: 10.7717/peerj.21692 (PMC13564400; doi:10.7717/peerj.21692)
Supplement: Supplementary file 7 [file peerj-14-21692-s007.docx]

**Supplemental Table 7.** GBIF records of *Arctium tomentum* from Pyrenees dismissed from the dataset. Region: Iberian Peninsula (IB), Balearic Islands (BA), Pyrenees (P).

| **Region** | **Locality** | **Coordinates** | **Date** | **Basis of record** | **GBIF id** | **reason for dismissal** |
| --- | --- | --- | --- | --- | --- | --- |
| IB | Alicante | 38°38′36.41″N, 0°02′52.34″E | 10/07/2019 | Human observation | 3952180344 | Observation without image |
| IB | Alicante | 38°38′36.41″N, 0°02′52.34″E | 10/07/2019 | Human observation | 3951808697 | Observation without image |
| IB | Alicante | 38°38′36.19″N, 0°02′52.34″E | 10/07/2019 | Human observation | 3951808696 | Observation without image |
| IB | Alicante | 38°38′36.41″N, 0°02′52.34″E | 10/07/2019 | Human observation | 3951671591 | Observation without image |
| BA | Mallorca | 39°44′24.00″N, 2°52′48.00″E | - | Human observation | 618142460 | Misinterpreted citation |
| BA | Mallorca | 39°46′12.00″N, 2°40′48.00″E | 18/07/1951 | Preserved specimen | 31969437 | Nomenclatural error |
| BA | Mallorca | 39°49′48.01″N, 2°53′24.00″E | 05/05/1944 | Preserved specimen | 31969435 | Nomenclatural error |
| BA | Mallorca | 39°46′47.64″N, 2°41′22.08″E | 15/08/1991 | Preserved specimen | 1265137633 | Only fruits |
| IB | Barcelona | 41°54′24.76″N, 1°42′18.29″E | 18/07/2024 | Human observation | 5110126481 | Image without diagnostic traits |
| IB | Cantabria | 42°57′40.55″N, 4°10′02.90″W | 28/05/2022 | Human observation | 3949855085 | Missidentification |
| IB | Girona | 42°23′38.35″N, 1°52′57.38″E | 17/07/2024 | Human observation | 5110323307 | Image without diagnostic traits |
| IB | Málaga | 37°04′38.40″N, 4°22′49.39″W | 22/05/2024 | Human observation | 5110220931 | Image without diagnostic traits |
| IB | Vizcaya | 43°15′39.83″N, 2°47′43.79″W | 24/04/2024 | Human observation | 5109940136 | Image without diagnostic traits |
| P | Ariège | 42°48′49.20″N, 1°15′32.98″E | 16/07/2023 | Human observation | 5109745280 | Missidentification |
| P | Bagnères-de-Bigorre | 42°50′41.32″N, 0°24′38.90″E | 22/07/2024 | Human observation | 5110186628 | Image without diagnostic traits |
| P | Oloron-Sainte-Marie | 42°55′38.02″N, 0°37′59.79″W | 31/07/2023 | Human observation | 5109827124 | Image without diagnostic traits |
| P | Bayonne | 43°07′51.24″N, 1°14′19.79″W | - | Human observation | 1119305142 | Not verifiable |
| IB | Coimbra | 40°12′18.33″N, 8°25′20.93″W | 17/09/1953 | Human observation | 4867469898 | Cultivated |
| IB | Coimbra | 40°12′18.33″N, 8°25′20.93″W | 07/09/1956 | Human observation | 4867457757 | Cultivated |
| IB | Coimbra | 40°12′18.33″N, 8°25′20.93″W | 18/08/1959 | Human observation | 4867479242 | Cultivated |
| IB | Coimbra | 40°12′18.33″N, 8°25′20.93″W | 06/09/1960 | Human observation | 4867406606 | Cultivated |
| IB | Coimbra | 40°12′18.33″N, 8°25′20.93″W | 24/08/1961 | Human observation | 4867405360 | Cultivated |
| IB | Coimbra | 40°12′18.33″N, 8°25′20.93″W | 06/09/1962 | Human observation | 4867402278 | Cultivated |
| IB | Coimbra | 40°12′18.33″N, 8°25′20.93″W | 13/08/1963 | Human observation | 4867418171 | Cultivated |
| IB | Coimbra | 40°12′18.33″N, 8°25′20.93″W | 11/09/1972 | Human observation | 4867486716 | Cultivated |
| IB | Coimbra | 40°12′18.33″N, 8°25′20.93″W | 25/08/1973 | Human observation | 4867428408 | Cultivated |
| IB | Coimbra | 40°12′18.33″N, 8°25′20.93″W | 21/08/1975 | Human observation | 4867435354 | Cultivated |
| IB | Coimbra | 40°12′18.33″N, 8°25′20.93″W | 31/08/1976 | Human observation | 4867500890 | Cultivated |
