# Supplementary material for: Evaluating three alternative scenarios for the origin of a disjunct Arctium tomentosum population in the Pyrenees
Source: PeerJ. 2026 Sep 7;14:e21692. doi: 10.7717/peerj.21692 (PMC13564400; doi:10.7717/peerj.21692)
Supplement: Supplementary file 8 [file peerj-14-21692-s008.docx]

**Supplemental Table 8.** Comparative analysis of repetitive elements in *Arctium* accessions. Geographic origin: Pyrenees (P), Slovenian Alps (SA), Alps (A).

|  | ***A. tomentosum* P** | | ***A. tomentosum* SA** | | ***A. tomentosum* A** | | ***A. lappa* P** | | ***A. lappa* A** | | ***A. minus* P** | | ***A. minus* A** | | ***A. nemorosum* A** | |
| --- | --- | --- | --- | --- | --- | --- | --- | --- | --- | --- | --- | --- | --- | --- | --- | --- |
| **Genome size (Mbp)** | 3804.42 | | 3618.6 | | 3618.6 | | 4078.26 | | 4078.26 | | 4577.04 | | 4577.04 | | 4694.4 | |
|  | **Genome proportion** | **Genome weight** | **Genome proportion** | **Genome weight** | **Genome proportion** | **Genome weight** | **Genome proportion** | **Genome weight** | **Genome proportion** | **Genome weight** | **Genome proportion** | **Genome weight** | **Genome proportion** | **Genome weight** | **Genome proportion** | **Genome weight** |
|  | **[%]** | **Mbp** | **[%]** | **Mbp** | **[%]** | **Mbp** | **[%]** | **Mbp** | **[%]** | **Mbp** | **[%]** | **Mbp** | **[%]** | **Mbp** | **[%]** | **Mbp** |
| **Retrotransposons**  **Ty1/copia** |  |  |  |  |  |  |  |  |  |  |  |  |  |  |  |  |
| SIRE | 32.53 | 1237.46 | 32.15 | 1163.55 | 35.45 | 1282.82 | 32.02 | 1305.88 | 35.14 | 1432.94 | 35.95 | 1645.34 | 38.47 | 1760.77 | 40.22 | 1888.3 |
| Angela | 0.6 | 22.87 | 0.5 | 17.98 | 0.58 | 21.13 | 0.6 | 24.35 | 0.54 | 21.87 | 0.57 | 26.01 | 0.61 | 27.84 | 0.64 | 30.09 |
| TAR | 0.66 | 25.2 | 0.69 | 25.13 | 0.66 | 23.76 | 0.7 | 28.69 | 0.64 | 26.16 | 0.73 | 33.4 | 0.62 | 28.24 | 0.63 | 29.54 |
| Bianca | 1.13 | 42.9 | 0.77 | 27.7 | 0.78 | 28.22 | 0.83 | 33.77 | 0.75 | 30.39 | 0.84 | 38.55 | 0.99 | 45.35 | 0.93 | 43.45 |
| Ale | 0.44 | 16.73 | 0.42 | 15.25 | 0.4 | 14.56 | 0.48 | 19.53 | 0.5 | 20.52 | 0.37 | 16.73 | 0.3 | 13.94 | 0.32 | 14.83 |
| Ivana | 0.01 | 0.54 | 0.06 | 2.03 | 0 | 0 | 0.02 | 0.95 | 0 | 0 | 0.01 | 0.67 | 0.01 | 0.65 | 0 | 0 |
| Tork | 0.35 | 13.5 | 0.27 | 9.66 | 0.23 | 8.47 | 0.28 | 11.29 | 0.24 | 9.8 | 0.3 | 13.95 | 0.36 | 16.68 | 0.38 | 17.77 |
| Other | 0 | 0 | 0 | 0 | 0 | 0 | 0 | 0 | 0.13 | 5.47 | 0.07 | 3.14 | 0 | 0 | 0 | 0 |
| **Total** | **35.73** | **1359.21** | **34.86** | **1261.3** | **38.11** | **1378.96** | **34.93** | **1424.45** | **37.94** | **1547.15** | **38.84** | **1777.79** | **41.37** | **1893.47** | **43.11** | **2023.97** |
|  |  |  |  |  |  |  |  |  |  |  |  |  |  |  |  |  |
| **Ty3/Gypsy** |  |  |  |  |  |  |  |  |  |  |  |  |  |  |  |  |
| Tekay | 7.5 | 285.47 | 7.3 | 264.23 | 6.9 | 249.5 | 7.15 | 291.65 | 7.69 | 313.48 | 6.99 | 319.95 | 4.97 | 227.46 | 5.59 | 262.36 |
| Athila | 1.48 | 56.3 | 1.52 | 55.12 | 1.46 | 52.66 | 1.36 | 55.3 | 1.4 | 56.99 | 1.18 | 54.04 | 1.23 | 56.49 | 1.28 | 59.86 |
| CRM | 0.11 | 4.3 | 1.05 | 38.05 | 0.09 | 3.18 | 0.17 | 7.08 | 0.18 | 7.23 | 0.13 | 5.94 | 0.08 | 3.66 | 0.2 | 9.3 |
| Retand | 0.81 | 30.65 | 0.82 | 29.78 | 0.82 | 29.7 | 0.9 | 36.75 | 0.9 | 36.8 | 0.85 | 38.98 | 0.65 | 29.96 | 0.76 | 35.73 |
| Other | 0 | 0 | 0 | 0 | 0 | 0 | 0 | 0 | 0.08 | 3.21 | 0.12 | 5.31 | 0.03 | 1.37 | 0.03 | 1.41 |
| **Total** | **9.9** | **376.73** | **10.7** | **387.18** | **9.26** | **335.04** | **9.58** | **390.79** | **10.24** | **417.71** | **9.27** | **424.23** | **6.97** | **318.93** | **7.85** | **368.66** |
|  |  |  |  |  |  |  |  |  |  |  |  |  |  |  |  |  |
| **Unclassified LTR** | 5.43 | 206.54 | 5.22 | 188.94 | 5.9 | 213.61 | 5.36 | 218.8 | 6 | 244.64 | 6.62 | 303.22 | 6.81 | 311.71 | 6.69 | 313.87 |
|  |  |  |  |  |  |  |  |  |  |  |  |  |  |  |  |  |
| **Pararetrovirus** | 0.05 | 2.07 | 0.06 | 2.02 | 0.01 | 0.47 | 0.05 | 2.17 | 0 | 0 | 0.11 | 4.88 | 0.07 | 3.22 | 0.08 | 3.84 |
|  |  |  |  |  |  |  |  |  |  |  |  |  |  |  |  |  |
| **LINE** | 0.03 | 1.27 | 0.03 | 1.13 | 0.03 | 0.91 | 0.03 | 1.34 | 0.04 | 1.58 | 0.05 | 2.47 | 0.03 | 1.45 | 0.03 | 1.28 |
|  |  |  |  |  |  |  |  |  |  |  |  |  |  |  |  |  |
| **DNA transposons** |  |  |  |  |  |  |  |  |  |  |  |  |  |  |  |  |
| TIR/Enspm_CACTA | 0.33 | 12.61 | 0.33 | 11.89 | 0.3 | 10.88 | 0.3 | 12.33 | 0.24 | 9.98 | 0.23 | 10.47 | 0.26 | 11.89 | 0.34 | 15.99 |
| TIR/MuDR-Mutator | 0.08 | 2.87 | 0.48 | 17.43 | 0.42 | 15.13 | 0.52 | 21.17 | 0.44 | 17.97 | 0.55 | 25.19 | 0.3 | 13.83 | 0.36 | 17.06 |
| TIR/haT | 0.23 | 8.79 | 0.16 | 5.96 | 0.13 | 4.62 | 0.09 | 3.84 | 0.08 | 3.32 | 0.19 | 8.64 | 0.05 | 2.5 | 0.32 | 15 |
| TIR/PIF_Harbinger | 0.05 | 1.85 | 0.1 | 3.51 | 0 | 0 | 0.07 | 2.79 | 0 | 0 | 0.17 | 7.8 | 0.11 | 5.14 | 0.11 | 5.28 |
| **Total** | **0.69** | **26.12** | **1.07** | **38.79** | **0.85** | **30.63** | **0.98** | **40.13** | **0.77** | **31.27** | **1.14** | **52.1** | **0.73** | **33.37** | **1.14** | **53.33** |
|  |  |  |  |  |  |  |  |  |  |  |  |  |  |  |  |  |
| **Helitron** | 0.27 | 10.18 | 0.36 | 13.2 | 0.1 | 3.44 | 0.31 | 12.45 | 0.05 | 1.95 | 0.28 | 12.6 | 0.64 | 29.52 | 0.11 | 4.97 |
|  |  |  |  |  |  |  |  |  |  |  |  |  |  |  |  |  |
| **Tandem repeats** |  |  |  |  |  |  |  |  |  |  |  |  |  |  |  |  |
| rDNA | 1.26 | 47.95 | 1.55 | 56.19 | 0.7 | 25.19 | 1.26 | 51.56 | 0.52 | 21.1 | 1.12 | 51.26 | 0.66 | 30.2 | 0.49 | 22.86 |
| Satellite | 3.03 | 115.34 | 1.02 | 36.97 | 2.29 | 83 | 1.55 | 63.16 | 1.08 | 43.86 | 0.87 | 40.05 | 1.11 | 50.85 | 0.9 | 42.27 |
|  |  |  |  |  |  |  |  |  |  |  |  |  |  |  |  |  |
| **Unclassified** | 6.8 | 258.77 | 7.52 | 272.06 | 7.1 | 256.96 | 8.73 | 355.87 | 9.3 | 379.39 | 6.95 | 318 | 8.4 | 384.28 | 7.75 | 363.96 |
| **Unclassified small repeats (%<0.01)** | 13.91 | 529.28 | 14.08 | 509.64 | 12.63 | 456.97 | 13.8 | 562.8 | 12.6 | 513.77 | 12.92 | 591.5 | 12.21 | 558.92 | 12.08 | 567.13 |
| **Single copy** | 22.89 | 870.96 | 23.52 | 851.18 | 23.03 | 833.41 | 23.41 | 954.74 | 21.48 | 875.84 | 21.82 | 998.94 | 21 | 961.13 | 19.77 | 928.26 |
