# Supplementary material for: Evaluating three alternative scenarios for the origin of a disjunct Arctium tomentosum population in the Pyrenees
Source: PeerJ. 2026 Sep 7;14:e21692. doi: 10.7717/peerj.21692 (PMC13564400; doi:10.7717/peerj.21692)
Supplement: Supplementary file 9 [file peerj-14-21692-s009.docx]

**Supplemental Table 9**. Pairwise R² values from linear regressions comparing estimated repeat cluster copy numbers across individuals. Sample provenance: Pyrenees (P), Alps (A), Slovenian Alps (SA) (see Table S3). dat derived from Table S8.

| **Taxon** | ***A. minus* P** | ***A. minus* A** | ***A. nemorosum* A** | ***A. lappa* P** | ***A. lappa* A** | ***A. tomentosum* A** | ***A. tomentosum* SA** | ***A. tomentosum* P** |
| --- | --- | --- | --- | --- | --- | --- | --- | --- |
| ***A. minus* P** | 1.000 | 0.979 | 0.986 | 0.981 | 0.973 | 0.964 | 0.96 | 0.958 |
| ***A. minus* A** | 0.979 | 1.000 | 0.996 | 0.950 | 0.949 | 0.944 | 0.927 | 0.925 |
| ***A. nemorosum* A** | 0.986 | 0.996 | 1.000 | 0.957 | 0.958 | 0.956 | 0.932 | 0.929 |
| ***A. lappa* P** | 0.981 | 0.950 | 0.957 | 1.000 | 0.986 | 0.975 | 0.985 | 0.984 |
| ***A. lappa* A** | 0.973 | 0.949 | 0.958 | 0.986 | 1.000 | 0.978 | 0.974 | 0.968 |
| ***A. tomentosum* A** | 0.964 | 0.944 | 0.956 | 0.975 | 0.978 | 1.000 | 0.978 | 0.976 |
| ***A. tomentosum* SA** | 0.96 | 0.927 | 0.932 | 0.985 | 0.974 | 0.978 | 1.000 | 0.998 |
| ***A. tomentosum* P** | 0.958 | 0.925 | 0.929 | 0.984 | 0.968 | 0.976 | 0.998 | 1.000 |
