# Supplementary material for: Evaluating three alternative scenarios for the origin of a disjunct Arctium tomentosum population in the Pyrenees
Source: PeerJ. 2026 Sep 7;14:e21692. doi: 10.7717/peerj.21692 (PMC13564400; doi:10.7717/peerj.21692)
Supplement: Supplementary file 10 [file peerj-14-21692-s010.docx]

**Supplemental Table 10.** Mean differences in functional traits between *Arctium minus* and *A. tomentosum*. Mean is based on 27 terminal fresh capitula for *A. minus* and on 25 for *A. tomentosum*.

| **Variable mm** | ***A. minus* mean ± SD^1^** | ***A. tomentosum* mean ± SD** | **W^2^** | ***p*-value** |
| --- | --- | --- | --- | --- |
| Capitulum height | 18.96 **±** 1.88 | 23.39 **±** 1.45 | 12 | **2.64e-09** |
| Involucre diametre | 16.08 **±** 2.67 | 16.56 **±** 2.37 | 354 | 0.769 |
| Involucre height | 12.19 **±** 1.83 | 13.18 **±** 1.35 | 242 | 0.082 |
| Bract number | 275.41 **±** 24.19 | 205.04 **±** 22.67 | 674.5 | **7.09E-10** |
| Flower number | 50.26 **±** 10.35 | 52.96 **±** 4.32 | 311.5 | 0.64 |

**^1^**Standard Deviation. **^2^**Value of Wilcoxon test statistic.
