# Supplementary material for: Evaluating three alternative scenarios for the origin of a disjunct Arctium tomentosum population in the Pyrenees
Source: PeerJ. 2026 Sep 7;14:e21692. doi: 10.7717/peerj.21692 (PMC13564400; doi:10.7717/peerj.21692)
Supplement: Supplementary file 11 [file peerj-14-21692-s011.docx]

**Supplemental Table 11.** Variance partitioning for the random-effects structure across fruit categories. Analyses were performed under two alternative assumptions: excluding the discrepancy between flowers and fruits, indicated in the Category column as “without”, or treating this discrepancy as fully predated fruits, indicated as “with”.

| **Category** | **year** | **pop** | **pop:year** |
| --- | --- | --- | --- |
| Well-formed | 100.00% | 0.00 | 0.00 |
| Predated without | 9.55% | 90.45% | 0.00 |
| Predated with | 29.54% | 70.46% | 0.00 |
| Aborted | 0.00 | 0.00 | 0.00 |
