# Supplementary material for: Evaluating three alternative scenarios for the origin of a disjunct Arctium tomentosum population in the Pyrenees
Source: PeerJ. 2026 Sep 7;14:e21692. doi: 10.7717/peerj.21692 (PMC13564400; doi:10.7717/peerj.21692)
Supplement: Supplementary file 12 [file peerj-14-21692-s012.docx]

**Supplemental Table 12.** Model fit comparison and diagnostic statistics for GLMMs fitted to well-formed, aborted, and predated fruits; for the last variable, analyses were performed under two alternative assumptions: excluding the discrepancy between flowers and fruits, indicated in the Model column as “without”, or treating this discrepancy as fully predated fruits, indicated as “with”. Significant *p*-values are highlighted in bold.

| **Model** | **AIC^1^ (binomial)** | **AIC^1^ (beta-binomial)** | **Binomial overdispersion *p*^2^** | **DHARMa uniformity *p*^3^** | **DHARMa dispersion *p*^4^** | **DHARMa zeroInfl p^5^** | **LRT species χ^2^ ^6^** | **LRT species *p*^7^** |
| --- | --- | --- | --- | --- | --- | --- | --- | --- |
| Well-formed | 2,102.30860 | 857.07720 | **0.00000** | 0.82260 | 0.28800 | 0.53600 | 100.28640 | **0.00000** |
| Predated “without” | 1,506.29950 | 852.07830 | 0.58400 | 0.54550 | 0.38400 | 0.66400 | 8.19640 | **0.00420** |
| Predated “with” | 1,596.09600 | 885.50800 | 0.32800 | 0.38967 | 0.95200 | 0.84000 | 7.69924 | **0.00552** |
| Aborted | 1,797.37110 | 882.40450 | **0.00000** | 0.45190 | 0.48800 | 0.11200 | 12.22250 | **0.00050** |

¹Akaike information criterion for the binomial model fitted with the same fixed and random structure as the final model; lower values indicate better relative fit. ^2^*p*-value testing whether the variance in the data exceeds that expected under a standard binomial model. < 0.05 values indicate overdispersion and support the use of a beta-binomial distribution. ^3^Simulation-based *p*-value of whether residuals from the fitted beta-binomial model follow the expected uniform distribution. ^4^Simulation-based *p*-value of whether the fitted beta-binomial model adequately captures the observed dispersion. ^5^simulation-based *p*-value of whether the fitted beta-binomial model shows an excess of zeros relative to model expectations. ^6^chi-square statistic from the likelihood ratio test comparing models with and without species as a fixed effect; larger values indicate a stronger contribution of species to explaining variation in the response. ^7^*p*-value associated with the likelihood ratio test for the species effect.
