# Supplementary material for: Evaluating three alternative scenarios for the origin of a disjunct Arctium tomentosum population in the Pyrenees
Source: PeerJ. 2026 Sep 7;14:e21692. doi: 10.7717/peerj.21692 (PMC13564400; doi:10.7717/peerj.21692)
Supplement: Supplementary file 13 [file peerj-14-21692-s013.docx]

**Supplemental Table 13.** Fixed-effect estimates from GLMMs for well-formed, predated and aborted fruits. Species was included as a fixed effect, with *A. minus* used as the reference level. Analyses were performed under two alternative assumptions: excluding the discrepancy between flowers and fruits, indicated in the Model column as “without”, or treating this discrepancy as fully predated fruits, indicated as “with”. Significant *p*-values are highlighted in bold.

| **Model** | **Term** | **Estimate (logit)^1^** | **SE^2^** | **Z^3^** | ***p*-value^4^** | **CI lower (2.5%)^5^** | **CI upper (97.5%)^5^** |
| --- | --- | --- | --- | --- | --- | --- | --- |
| Well-formed without | (Intercept) | -1.73570 | 0.32510 | -5.33950 | **0.00000** | -2.37290 | -1.09860 |
| Well-formed without | Species | 2.49040 | 0.21690 | 11.48140 | **0.00000** | 2.06530 | 2.91550 |
| Well-formed with | (Intercept) | -1.73574 | 0.32508 | -5.33946 | **0.00000** | -2.37288 | -1.09860 |
| Well-formed with | Species | 2.49039 | 0.21691 | 11.48145 | **0.00000** | 2.06526 | 2.91552 |
| Predated without | (Intercept) | 0.22070 | 0.26950 | 0.81900 | 0.41280 | -0.30750 | 0.74890 |
| Predated without | Species | -2.60860 | 0.54060 | -4.82530 | **0.00000** | -3.66820 | -1.54910 |
| Predated with | (Intercept) | 0.48438 | 0.31979 | 1.51468 | 0.12985 | -0.14240 | 1.11115 |
| Predated with | Species | -2.43625 | 0.52906 | -4.60489 | **0.00000** | -3.47318 | -1.39932 |
| Aborted without | (Intercept) | -1.08670 | 0.11240 | -9.67090 | **0.00000** | -1.30700 | -0.86650 |
| Aborted without | Species | -0.61530 | 0.17550 | -3.50610 | **0.00050** | -0.95930 | -0.27130 |
| Aborted with | (Intercept) | -1.08674 | 0.11237 | -9.67088 | **0.00000** | -1.30698 | -0.86649 |
| Aborted with | Species | -0.61531 | 0.17550 | -3.50611 | **0.00046** | -0.95927 | -0.27134 |

^1^Logit: effect estimate on the logit scale, expressing the direction and magnitude of the relationship relative to the reference category. ²Standard Error. ³Wald statistic used to test whether the estimated effect differs from zero; higher absolute values reflect stronger evidence of an effect.^4^Significance level associated with the Wald test.^5^Range of values compatible with the estimated effect at the 95% confidence level; intervals excluding zero support a non-null effect.
