# Supplementary material for: Evaluating three alternative scenarios for the origin of a disjunct Arctium tomentosum population in the Pyrenees
Source: PeerJ. 2026 Sep 7;14:e21692. doi: 10.7717/peerj.21692 (PMC13564400; doi:10.7717/peerj.21692)
Supplement: Supplementary file 14 [file peerj-14-21692-s014.docx]

**Supplemental Table 14.** Empirical and model-based differences in lower-to-fruit rates between *Arctium minus* and *A. tomentosum*. Analyses were performed under two alternative assumptions: excluding the discrepancy between flowers and fruits, indicated in the Category column as “without”, or treating this discrepancy as fully predated fruits, indicated as “with”. Significant *p*-values are highlighted in bold.

| **Category** | **Species** | **n** | **Observed mean (%) ± 95% CI¹** | **Model mean (%) ± 95% CI²** | **W^3^** | ***p*-value/ *p*-model^4^** |
| --- | --- | --- | --- | --- | --- | --- |
| Well-formed | *A. minus* | 74 | 15.2 (10.4–19.9) | 15 (8.5–25) | 259.5 | **9.97e-18 / 1.64e-30** |
|  | *A. tomentosum* | 57 | 69.8 (63.5–76.1) | 68 (53.1–80) |  |  |
| Predated “without” | *A. minus* | 74 | 53.8 (48.7–58.8) | 55.5 (42.4–67.9) | 3991 | **7.69e-18 / 1.40e-06** |
|  | *A. tomentosum* | 57 | 11.3 (7.5–15) | 8.4 (3.4–19.3) |  |  |
| Predated “with” | *A. minus* | 74 | 61 (55.5–66.5) | 61.9 (46.4–75.2) | 3972.5 | **9.92e-18 / 4.13e-06** |
|  | *A. tomentosum* | 57 | 14.5 (10.3–18.6) | 12.4 (5–27.7) |  |  |
| Aborted | *A. minus* | 74 | 23.9 (20–27.7) | 25.2 (21.3–29.6) | 2980.5 | **5.26e-05 / 4.55e-04** |
|  | *A. tomentosum* | 57 | 15.7 (10.3–21.2) | 15.4 (12–19.5) |  |  |

^1^Observed mean with empirical 95% confidence interval. ^2^Model-predicted mean with 95% confidence interval. ^3^Value of Wilcoxon test statistic. ^4^Empirical-based species-contrast *p*-value / model-based species-contrast *p*-value.
