# Supplementary material for: Evaluating three alternative scenarios for the origin of a disjunct Arctium tomentosum population in the Pyrenees
Source: PeerJ. 2026 Sep 7;14:e21692. doi: 10.7717/peerj.21692 (PMC13564400; doi:10.7717/peerj.21692)
Supplement: Supplementary file 15 [file peerj-14-21692-s015.docx]

**Supplemental Table 15.** Non-parametric pairwise comparisons of conversion rates in *A. minus* and *A. tomentosum*. The multiple comparison based on the Kruskal–Wallis test for August 2022 among the OH 728, OH 775 and OH777 *A. minus* populations was omitted, as the mean and standard deviation for each population in that month and year are already provided, and the test was not significant. Significant *p*-values are highlighted in bold. Analyses were performed under two alternative assumptions in predated Response column: excluding the discrepancy between flowers and fruits, indicated in the Coding column as “without”, or treating this discrepancy as fully predated fruits, indicated as “with”.

| **Block** | **Response** | **Coding** | **Group 1** | **n** | **Mean ± SD^1^** | **Group 2** | **n** | **Mean ± SD^1^** | **W^2^** | ***p*-value** |
| --- | --- | --- | --- | --- | --- | --- | --- | --- | --- | --- |
| Grouped annual comparisons | well-formed | — | *A. tomentosum* (pooled) 2022 | 26 | 0.646 ± 0.246 | *A. minus* (pooled) 2022 | 43 | 0.058 ± 0.087 | 1075 | **1.39e-09** |
| Grouped annual comparisons | aborted | — | *A. tomentosum* (pooled) 2022 | 26 | 0.190 ± 0.230 | *A. minus* (pooled) 2022 | 43 | 0.215 ± 0.139 | 412 | 0.2408 |
| Grouped annual comparisons | predated | without | *A. tomentosum* (pooled) 2022 | 26 | 0.123 ± 0.148 | *A. minus* (pooled) 2022 | 43 | 0.628 ± 0.182 | 28 | **5.21e-10** |
| Grouped annual comparisons | predated | with | *A. tomentosum* (pooled) 2022 | 26 | 0.165 ± 0.164 | *A. minus* (pooled) 2022 | 43 | 0.727 ± 0.184 | 21 | **3.09e-10** |
| Grouped annual comparisons | well-formed | — | *A. tomentosum* (pooled) 2023 | 31 | 0.742 ± 0.226 | *A. minus* (pooled) 2023 | 31 | 0.281 ± 0.251 | 870.5 | **2.26e-07** |
| Grouped annual comparisons | aborted | — | *A. tomentosum* (pooled) 2023 | 31 | 0.130 ± 0.183 | *A. minus* (pooled) 2023 | 31 | 0.271 ± 0.193 | 232.5 | **0.0054** |
| Grouped annual comparisons | predated | without | *A. tomentosum* (pooled) 2023 | 31 | 0.104 ± 0.136 | *A. minus* (pooled) 2023 | 31 | 0.412 ± 0.204 | 87 | **1.44e-07** |
| Grouped annual comparisons | predated | with | *A. tomentosum* (pooled) 2023 | 31 | 0.128 ± 0.150 | *A. minus* (pooled) 2023 | 31 | 0.448 ± 0.204 | 96.5 | **3.38e-07** |
| Grouped annual comparisons | well-formed | — | *A. minus* (pooled) 2022 | 43 | 0.058 ± 0.087 | *A. minus* (pooled) 2023 | 31 | 0.281 ± 0.251 | 293 | **1.27e-04** |
| Grouped annual comparisons | aborted | — | *A. minus* (pooled) 2022 | 43 | 0.215 ± 0.139 | *A. minus* (pooled) 2023 | 31 | 0.271 ± 0.193 | 572.5 | 0.4202 |
| Grouped annual comparisons | predated | without | *A. minus* (pooled) 2022 | 43 | 0.628 ± 0.182 | *A. minus* (pooled) 2023 | 31 | 0.412 ± 0.204 | 1053 | **0.0001** |
| Grouped annual comparisons | predated | with | *A. minus* (pooled) 2022 | 43 | 0.727 ± 0.184 | *A. minus* (pooled) 2023 | 31 | 0.448 ± 0.204 | 1117.5 | **2.93e-06** |
| Grouped annual comparisons | well-formed | — | *A. tomentosum* (pooled) 2022 | 26 | 0.646 ± 0.246 | *A. tomentosum* (pooled) 2023 | 31 | 0.742 ± 0.226 | 292 | 0.1500 |
| Grouped annual comparisons | aborted | — | *A. tomentosum* (pooled) 2022 | 26 | 0.190 ± 0.230 | *A. tomentosum* (pooled) 2023 | 31 | 0.130 ± 0.183 | 493.5 | 0.2636 |
| Grouped annual comparisons | predated | without | *A. tomentosum* (pooled) 2022 | 26 | 0.123 ± 0.148 | *A. tomentosum* (pooled) 2023 | 31 | 0.104 ± 0.136 | 440 | 0.5433 |
| Grouped annual comparisons | predated | with | *A. tomentosum* (pooled) 2022 | 26 | 0.165 ± 0.164 | *A. tomentosum* (pooled) 2023 | 31 | 0.128 ± 0.150 | 480 | 0.2982 |
| Spatial within species | well-formed | — | *A. minus* OH 777 August 2022 | 8 | 0.079 ± 0.112 | *A. minus* OH 728 August 2022 | 27 | 0.059 ± 0.091 | 124 | 0.6852 |
| Spatial within species | aborted | — | *A. minus* OH 777 August 2022 | 8 | 0.231 ± 0.101 | *A. minus* OH 728 August 2022 | 27 | 0.231 ± 0.145 | 117 | 0.7726 |
| Spatial within species | predated | without | *A. minus* OH 777 August 2022 | 8 | 0.630 ± 0.184 | *A. minus* OH 728 August 2022 | 27 | 0.588 ± 0.181 | 130 | 0.4761 |
| Spatial within species | predated | with | *A. minus* OH 777 August 2022 | 8 | 0.690 ± 0.203 | *A. minus* OH 728 August 2022 | 27 | 0.710 ± 0.188 | 102 | 0.8289 |
| Spatial within species | well-formed | — | *A. minus*OH 777 August 2022 | 8 | 0.079 ± 0.112 | *A. minus* OH 779 August 2022 | 8 | 0.034 ± 0.025 | 38 | 0.6852 |
| Spatial within species | aborted | — | *A. minus* OH 777 August 2022 | 8 | 0.231 ± 0.101 | *A. minus* OH 779 August 2022 | 8 | 0.147 ± 0.145 | 47.5 | 0.2528 |
| Spatial within species | predated | without | *A. minus* OH 777 August 2022 | 8 | 0.630 ± 0.184 | *A. minus* OH 779 August 2022 | 8 | 0.763 ± 0.128 | 16 | 0.1627 |
| Spatial within species | predated | with | *A. minus* OH 777e August 2022 | 8 | 0.690 ± 0.203 | *A. minus* OH 779 August 2022 | 8 | 0.820 ± 0.134 | 14 | 0.1454 |
| Spatial within species | well-formed | — | *A. minus* OH 728 August 2022 | 27 | 0.059 ± 0.091 | *A. minus* OH 779 August 2022 | 8 | 0.034 ± 0.025 | 106 | 0.9514 |
| Spatial within species | aborted | — | *A. minus* OH 728 August 2022 | 27 | 0.231 ± 0.145 | *A. minus* OH 779 August 2022 | 8 | 0.147 ± 0.145 | 152 | 0.2408 |
| Spatial within species | predated | without | *A. minus* OH 728 August 2022 | 27 | 0.588 ± 0.181 | *A. minus* OH 779 August 2022 | 8 | 0.763 ± 0.128 | 51 | 0.0582 |
| Spatial within species | predated | with | *A. minus* OH 728 August 2022 | 27 | 0.710 ± 0.188 | *A. minus* OH 779 August 2022 | 8 | 0.820 ± 0.134 | 69 | 0.2391 |
| Temporal within population | well-formed | — | *A. tomentosum* OH 775 July 2023 | 18 | 0.691 ± 0.247 | *A. tomentosum* OH 775 September 2023 | 11 | 0.851 ± 0.164 | 55 | 0.1390 |
| Temporal within population | aborted | — | *A. tomentosum* OH 775 July 2023 | 18 | 0.185 ± 0.224 | *A. tomentosum* OH 775 September 2023 | 11 | 0.041 ± 0.035 | 137.5 | 0.2408 |
| Temporal within population | predated | without | *A. tomentosum* OH 775 July 2023 | 18 | 0.117 ± 0.153 | *A. tomentosum* OH 775 September 2023 | 11 | 0.075 ± 0.118 | 119.5 | 0.4676 |
| Temporal within population | predated | with | *A. tomentosum* OH 775 July 2023 | 18 | 0.123 ± 0.153 | *A. tomentosum* OH 775 September 2023 | 11 | 0.107 ± 0.150 | 112 | 0.6198 |
| Temporal within population | well-formed | — | *A. minus* OH 777 August 2022 | 8 | 0.079 ± 0.112 | *A. minus* OH 777 August 2023 | 11 | 0.140 ± 0.091 | 25 | 0.1979 |
| Temporal within population | aborted | — | *A. minus* OH 777 August 2022 | 8 | 0.231 ± 0.101 | *A. minus* OH 777e August 2023 | 11 | 0.244 ± 0.181 | 40 | 0.7726 |
| Temporal within population | predated | without | *A. minus* OH 777 August 2022 | 8 | 0.630 ± 0.184 | *A. minus* OH 777 August 2023 | 11 | 0.575 ± 0.199 | 54 | 0.4761 |
| Temporal within population | predated | with | *A. minus* OH 777e August 2022 | 8 | 0.690 ± 0.203 | *A. minus* OH 777 August 2023 | 11 | 0.616 ± 0.181 | 57 | 0.3691 |
| Spatial within species | well-formed | — | *A. minus* OH 777e August 2023 | 11 | 0.140 ± 0.091 | *A. minus* OH 951 August 2023 | 20 | 0.359 ± 0.277 | 67.5 | 0.1500 |
| Spatial within species | aborted | — | *A. minus* OH 777e August 2023 | 11 | 0.244 ± 0.181 | *A. minus* OH 951 August 2023 | 20 | 0.286 ± 0.202 | 100 | 0.7726 |
| Spatial within species | predated | without | *A. minus* OH 777 August 2023 | 11 | 0.575 ± 0.199 | *A. minus* OH 951 August 2023 | 20 | 0.322 ± 0.145 | 187 | **0.0044** |
| Spatial within species | predated | with | *A. minus* OH 777 August 2023 | 11 | 0.616 ± 0.181 | *A. minus* OH 951 August 2023 | 20 | 0.355 ± 0.152 | 192 | 0.0021 |

^1^Standard Deviation. ^2^Value of Wilcoxon test statistic.
