# Supplementary material for: Evaluating three alternative scenarios for the origin of a disjunct Arctium tomentosum population in the Pyrenees
Source: PeerJ. 2026 Sep 7;14:e21692. doi: 10.7717/peerj.21692 (PMC13564400; doi:10.7717/peerj.21692)
Supplement: Supplementary file 16 [file peerj-14-21692-s016.docx]

**Supplemental Table 16.** Linear models relating flower production to well-formed fruits and predation to abortion relative to well-formed fruits under both coding schemes. Significant *p*-values are highlighted in bold.

| **Linear model** | **Species** | **Intercept** | **Slope** | ***p*-value** | **R²** | **n** |
| --- | --- | --- | --- | --- | --- | --- |
| Flowers to well formed fruits | *A. minus* | -1.0794 | 0.176807 | 0.191693 | 0.023557 | 74 |
| Flowers to well formed fruits | *A. tomentosum* | -40.469 | 1.458737 | **3.43e-05** | 0.270117 | 57 |
| Predation to abortion relative to well formed (log-ratio) without | *A. minus* | -0.5784 | 0.815317 | **1.20e-18** | 0.662407 | 74 |
| Predation to abortion relative to well formed (log-ratio) without | *A. tomentosum* | -1.34889 | 0.238319 | 0.050877 | 0.067554 | 57 |
| Predation to abortion relative to well formed (log-ratio) with | *A. minus* | -0.6702 | 0.810485 | **6.91e-19** | 0.667541 | 74 |
| Predation to abortion relative to well formed (log-ratio) with | *A. tomentosum* | -1.31989 | 0.297037 | **0.022193** | 0.091503 | 57 |
