# Supplementary material for: Evaluating three alternative scenarios for the origin of a disjunct Arctium tomentosum population in the Pyrenees
Source: PeerJ. 2026 Sep 7;14:e21692. doi: 10.7717/peerj.21692 (PMC13564400; doi:10.7717/peerj.21692)
Supplement: Supplementary file 17 [file peerj-14-21692-s017.docx]

**Supplemental Table 17**. Composition in percentage of insect assemblages per population and month for *A. minus* and *A. tomentosum.*  Data derived from Table S6.

| **Host information** | | | **N** | |  | **Diptera** | | **Lepidoptera** | **Hymenoptera** | | **Hemiptera** | | **Coleoptera** |
| --- | --- | --- | --- | --- | --- | --- | --- | --- | --- | --- | --- | --- | --- |
| **Taxon** | **Pop.** | **Month** | **Capitula** | **Insect ind.** | **Mean insect/cap**  **± SD^1^** | **Tephritidae larvae** | **Tephritidae adult** | ***Metzneria lappella***  **larvae** | **Pteromalidae** | ***Bracon* sp.** | ***Anthocoris nemorum*** | ***Orius laevigatus*** | ***Corticaria obscura*** |
| *A. minus* | OH 951 | August | 26 | 110 | 4.40 ± 2.31 | 69.37% | 8,11% | 2.70% | 13.51% | 5.51% | 0 | 0 | 0,90% |
|  | OH 777 |  | 12 | 96 | 8.00 ± 6.22 | 96.88% | 1,04% | 0 | 0 | 0 | 1,04% | 1,04% | 0 |
| *A. tomentosum* | OH 775 | July | 13 | 33 | 2.54 ± 2.65 | 36.36% | 0 | 57,57% | 0% | 6,01% | 0 | 0 | 0 |
|  |  | September | 8 | 25 | 3.13 ± 1.73 | 80.00% | 8,00% | 8,00% | 4.00% | 0 | 0 | 0 | 0 |

^1^Standard Deviation.
